# Exploring parental prenatal influences on child health: A multicohort study and data visualisation tool

**DOI:** 10.1371/journal.pmed.1005153

**Published:** 2026-07-23

**Authors:** Gemma C. Sharp, Deborah A. Lawlor, Kayleigh E. Easey, Sampurna Kundu, Ahmed Elhakeem, Edward Hone, Rosemary R. C. McEachan, Maria C. Magnus, Alexandra Havdahl, Caroline L. Relton

**Affiliations:** 1 School of Psychology, Faculty of Health and Life Sciences, University of Exeter, Exeter, United Kingdom; 2 MRC Integrative Epidemiology Unit at the University of Bristol, Bristol, United Kingdom; 3 Population Health Science, Bristol Medical School, University of Bristol, Bristol, United Kingdom; 4 School of Psychological Science, University of Bristol, Bristol, United Kingdom; 5 Research Software Engineers Group, University of Exeter, Exeter, United Kingdom; 6 Bradford Institute for Health Research, Bradford Teaching Hospitals National Health Service Foundation Trust, Bradford, United Kingdom; 7 Centre for Fertility and Health, Norwegian Institute of Public Health, Oslo, Norway; 8 Nic Waals Institute, Lovisenberg Diaconal Hospital, Oslo, Norway; 9 PsychGen Centre for Genetic Epidemiology and Mental Health, Division of Public Health and Prevention, Norwegian Institute of Public Health, Oslo, Norway; 10 Center for Genetic Epidemiology and Mental Health, Norwegian Institute of Public Health, Oslo, Norway; 11 PROMENTA Research Centre, Department of Psychology, University of Oslo, Oslo, Norway; 12 London School of Hygiene and Tropical Medicine, London, United Kingdom; Stellenbosch University, SOUTH AFRICA

## Abstract

**Background:**

The Developmental Origins of Health and Disease (DOHaD) hypothesis suggests that early life environmental exposures, especially during pregnancy, can impact long-term health. Research has largely relied on correlational evidence and has focussed on maternal factors, with less attention given to paternal, postnatal, and broader social determinants. This focus could complicate efforts to determine the most effective strategies for improving population health.

**Methods and findings:**

Using harmonised data across four population-based longitudinal cohort studies from the UK and Norway (births between 1991 and 2008), we took a systematic approach to explore associations of parental prenatal health behaviours (smoking, alcohol, and caffeine consumption) and low socioeconomic position (SEP) with 72 child health-related outcomes (e.g., related to body size and composition, cognitive function, mental health, blood pressure, allergy, etc.) from birth up to age 11. Where possible, cohort estimates were meta-analysed, yielding a maximum sample size of 232,139. We triangulated evidence of causality using different analytical approaches, including a Mendelian randomisation-informed approach using genetic risk scores, negative controls (maternal versus paternal and during versus post-pregnancy comparisons), and dose–response analyses. This comprehensive set of analyses generated more than 594,000 effect estimates. We developed a web app, ‘EPoCH Explorer’ to visualise and share our results in an accessible format. We did not find strong evidence for widespread or large effects of parental health behaviours on child health and wellbeing. Only 6% of analyses had a Cohen’s *D* value >0.2 and FDR-adjusted *P* < 0.05. In most analyses, the effect estimate was similar for mothers and partners, with 51% showing a larger effect for mothers and 49% for partners. Despite the lack of widespread associations, we found consistent evidence of association between maternal smoking and small for gestational age, higher childhood body mass index (BMI), depressive symptoms, and behavioural issues, while partner smoking was consistently associated with childhood BMI and social communication difficulties. Overall, we found stronger evidence of child outcomes being associated with low SEP than with health behaviours: 15% of results for low SEP had a Cohen’s *D* value >0.2 and FDR-*P* < 0.05, compared to 6% for parental smoking, 3% for alcohol, and 0.4% for caffeine. Sample sizes and statistical power varied across outcomes and there was limited ethnic diversity in our study samples.

**Conclusion:**

Our findings suggest that wider familial socioeconomic conditions may be a more important determinant of child health than specific parental health behaviours prenatally. Interventions to improve population health may be most effective if they target wider social inequalities, rather than individual behaviours and mothers specifically. We encourage researchers to use EPoCH Explorer to prioritise associations for exploration in their own datasets, thus enabling replication and cross-context comparison to validate and extend the generalisability of our findings.

## Author summary

**Why was this study done?:**

- Early life conditions are thought to shape long-term health, but most research has focussed on mothers during pregnancy and used observational data that can be hard to interpret.
- Less is known about the roles of fathers, post-pregnancy influences, and wider social circumstances such as family socioeconomic position.
- A clearer understanding of which early-life factors matter most is needed to guide effective public health strategies.

**What did the researchers do and find?:**

- Researchers analysed harmonised data from four large long-term cohort studies, including over 230,000 participants, to examine links between parental behaviours (smoking, alcohol, caffeine), socioeconomic position, and 72 child health outcomes up to age 11.
- They used multiple methods to strengthen causal interpretation.
- Only a small proportion of results suggested meaningful associations for parental behaviours (e.g., 6% for smoking), while stronger associations were found more consistently for low socioeconomic position (15%).

**What do these findings mean?:**

- The results suggest that broader family socioeconomic conditions may play a larger role in shaping child health than specific parental behaviours during pregnancy.
- Policies aiming to improve child health may have greater impact if they address social inequalities alongside, or instead of, focussing only on mothers and individual behaviours only.
- As the study relies on observational data and some of the analyses had missing information, some effects may have been missed. The participants were mostly white, and all living in the UK or Norway, so further research is needed in more diverse populations.

## Abstract 3

In this multi-cohort study, Gemma Sharp and colleagues analysed associations between parental behaviours and child health outcomes using four different cohorts, and generated a tool to visualize the results.

## Introduction

The Developmental Origins of Health and Disease (DOHaD) hypothesis posits that environmental exposures in early life, including those that occur prenatally, can affect long-term health [1]. Traditionally, the field has focussed on aspects of the maternal environment in and around pregnancy, including how a mother’s health behaviours might affect her fetus in utero (i.e., “maternal pregnancy effects”) [2]. Despite the fact that these behaviours are socially-patterned and likely to be correlated with paternal and postnatal exposures, there has been much less of a focus on these other types of exposure [2–4]. This makes it difficult to test (rather than merely reinforce) the DOHaD hypothesis, and to challenge implicit and deeply-held assumptions about the causal primacy of maternal pregnancy effects [2]. It also restricts our ability to disentangle specific causal effects from the wider system in which they occur and to understand whether efforts to improve offspring health would be most effective if they were directed towards wider social determinants rather than specific behaviours of individual parents.

In addition, current evidence used to support guidelines about changes to behaviour around pregnancy is often based on correlational rather than causal evidence [3,5,6]. Although the field necessarily relies largely on observational data, few studies have employed techniques that attempt to infer causal effects from such data. Even fewer have triangulated [7] evidence from multiple causal inference techniques to try to balance competing assumptions and improve confidence in causal conclusions. Finally, although copious prenatal (mostly maternal) exposure and offspring outcome combinations have been explored in the literature, sample sizes have often been small and differences in study design, populations, and definitions of variables can make direct comparisons and meta-analysis difficult [8–10].

The Exploring Prenatal influences on Childhood Health (EPoCH) study attempted to address these challenges. We took a systematic approach to explore associations between prenatal health behaviours (smoking, alcohol, and caffeine consumption) in both mothers and their partners, and multiple health outcomes of their children, across four longitudinal cohort studies with a combined maximum sample size of over 230,000. To enable exploration of causality, we also applied the principles of Mendelian randomisation (MR) by calculating associations between child health outcomes and parents’ genetic risk scores for each health behaviour [11], explored time- and dose- specific effects [12], and considered the equivalent exposure in the other parent, or outside of pregnancy, as a negative control [13]. Each of these causal inference methods has its own sources of bias, but the biases differ across approaches. To enhance confidence in our causal inferences, we undertook triangulation [7] to compare evidence from multiple methods wherever possible, reducing the impact of any single source of bias on our conclusions.

To help contextualise evidence for effects of the health behaviours of individual parents against effects of wider social influences on health, we also explored parental socioeconomic position (SEP) as an exposure.

We meta-analysed results from individual cohorts where possible, yielding results from over 594,000 analyses involving parental exposures at up to nine timepoints, 72 outcomes over four age ranges, and eight adjustment sets. We have made these data available to the research community through EPoCH Explorer, a web-app providing interactive and customisable data visualisations, interpretations, and downloads.

In this paper, we describe some high-level insights regarding the relative strength of evidence for effects of mother versus partners, and health behaviours versus socioeconomic factors. We illustrate how EPoCH Explorer can be used to visualise triangulated evidence and provide causal insights. For this illustration, we use the effect of maternal smoking on child externalising traits (i.e., disruptive, hyperactive, or aggressive behaviours [14]) as an example. Finally, we invite researchers to use EPoCH Explorer in their own studies, for example: to generate hypotheses for further detailed exploration of causality in their own datasets; to check for replication of their own findings in EPoCH cohorts; or to further increase sample size and generalisability through meta-analysis of EPoCH results with comparable results from their own datasets.

## Methods

### Cohorts

We used data from four cohorts: the Avon Longitudinal Study of Parents and Children (ALSPAC; United Kingdom, pregnancies in 1991–1992) [15,16], Born in Bradford (BiB; United Kingdom, pregnancies in 2007–2011) [17,18], Millennium Cohort Study (MCS; United Kingdom, pregnancies in 2000–2001) [19], and the Mother, Father, and Child Cohort Study (MoBa; Norway, pregnancies in 1999–2008) [20]. These cohorts were chosen because they collected data on most of the exposures/outcomes of interest, and had multiple follow-ups during childhood. More information about each cohort is provided in S1 File.

### Participants

Within each cohort, we excluded any children from multiple pregnancies (see Fig A in S1 File for sample sizes before and after exclusions). No exclusions were made based on any other variable, but all our analyses were complete case, meaning that parent-child pairs were excluded from analyses if they did not have complete data on the exposure, outcome, and covariates for any specific exposure-outcome analysis. Therefore, the sample size varies between different exposure-outcome associations. For all cohorts, partners were invited by the enrolled study mother. This person cannot always be assumed to be the biological father, and paternity information was not collected by every cohort. Therefore, we use the term ‘partner’ rather than ‘father’.

### Parental exposures

To maximise the relevance to public health policy, we focussed on parental health behaviours that are potentially modifiable during pregnancy: tobacco smoking, alcohol consumption, and caffeine consumption. Where data were available (summarised in S2 File), we derived each exposure variable for both mothers and partners. For each health behaviour, we derived several variables based on the nature and timing of exposure, with definitions harmonised across the four cohorts. These were: active smoking, passive smoke exposure, alcohol consumption, episodes of binge drinking, caffeine consumed from tea, coffee and cola (both individually and combined). We considered up to eight timepoints: preconception (in the three to six months before pregnancy), separately in each trimester of pregnancy, in the two years postnatally, at any time during pregnancy, and for active smoking only, early onset (started smoking before age 11) and ever in life. Where possible, we derived ordinal variables with four levels of exposure: heavy, moderate, light and none (for definitions, see S1 File). All other variables were binary (any versus no exposure), or for caffeine consumption only, continuous (mg/day).

In addition to health behaviours, we also explored parental SEP as an exposure class, indicative of the wider social determinants of health. We defined separate binary measures of SEP based on the highest education level achieved and current occupational social class where available. These variables compared the group with the lowest SEP (cohort specific—see S1 File) to all other groups as the reference.

### Genetic risk scores for parental exposures

We had access to genetic data for mothers in ALSPAC, BiB and MoBa, and partners (confirmed biological fathers in this instance) in MoBa. We calculated genetic risk scores (GRS) for parental smoking, alcohol, and coffee consumption to incorporate genetically informed analyses within a MR framework [21]. Specifically, GRS were used as proxies for these behaviours in reduced-form (genome-outcome) analyses. GRS were calculated using PLINK V1.9, using genetic variants (SNPs) identified in large scale genome-wide association studies (GWAS) of number of aggregated alcoholic drinks consumed per week [22], number of cups of coffee consumed per day [23], and four smoking-related traits: age of initiation or regular smoking, ever versus never been a regular smoker, cigarettes per day, smoking cessation (current versus former smoker) [22] (Tables A–F in S1 File). Scores were weighted by the effect estimates reported in the corresponding GWAS and converted to standard deviation units (*z* scores) to facilitate comparing effects across different traits. F-statistics for instrument strength—that is, the strength of the association between each GRS and the exposure it is intended to represent—are shown in Fig B in S1 File.

### Child outcomes and age stages

Where data were available (S2 File), we explored a broad range of child outcomes over five health domains: body size and adiposity; psychosocial and cognitive; immunological; serum biomarkers; and blood pressure. Outcomes were measured at up to six different timepoints in childhood (up to age 11) depending on the outcome and data availability in each cohort: at delivery, first year, age 1–2 years, age 3–4 years, age 5–7 years, age 8–11 years. In addition, for some outcomes (e.g., asthma) it was appropriate to develop a variable to describe the outcome occurring or being diagnosed “any time in childhood” (up to age 11). All continuous outcomes were converted to standard deviation units (z scores) so that they could be meta-analysed across different cohorts and visualised on the same scale. To facilitate interpretation of estimates, we also generated binary variables for birthweight, BMI and some psychosocial and cognitive outcomes, based on commonly used clinical thresholds. Outcome variables are described in detail in S1 File.

### Covariates

The definition and harmonisation method for all covariates is described in detail in S1 File.

Where data were available, we derived harmonised variables for potential confounders of the relationship between parental health behaviours and child health (based on the established definition that confounders must be known or plausible causes of both the exposure and outcome): parents’ ethnicity (Asian, Black, white, mixed or other); parents’ age at conception or delivery (years); parents’ highest education (ordered categories, as defined in each cohort); parents’ occupational social class (ordered categories, as defined in each cohort); mother’s parity (nulliparous versus parity ≥1).

We also generated harmonised variables for potential mediators (i.e., variables that potentially sit on the causal pathway between an exposure and an outcome) to include in a sensitivity analysis for some exposure-outcome combinations: gestational age at delivery (weeks); birthweight (kg); child’s caffeine consumption before age 2 (where available); child’s exposure to passive smoke before age 2 (where available).

Finally, all models were adjusted for sex (male, female) and age of the child at outcome measurement (years) to increase the precision of the estimate.

### Statistical analysis in each cohort

We conducted linear (for continuous outcomes) and logistic (for binary outcomes) multivariable regression models for each possible exposure/outcome combination, running up to eight models per exposure/outcome combination. These models and their rationales are outlined in Table 1; further details and the directed acyclic graphs (DAGs) are provided in S3 File. Where a cohort did not collect data on a particular covariate, it was omitted from the adjustment set for that cohort.

**Table 1:** Regression models that were run for each cohort.

Model number and name	Rationale	Adjustment set where the exposure is a health behaviour (i.e., smoking, alcohol, or caffeine)	Adjustment set where the exposure is socioeconomic position	Adjustment set where the exposure is a genetic risk score (GRS)
Model 1a: minimal—unadjusted for co-parent	Explores associations with maximum power and minimum selection, while still controlling for important covariates.	Child’s sex, child’s age at outcome measurement^*^, parent-of-interest’s ethnicity	Child’s sex, child’s age at outcome measurement^*^, parent-of-interest’s ethnicity	N/A
Model 1b: minimal—adjusted for co-parent	Additionally controls for the co-parent’s exposure	Model 1a + *co-parent’s exposure*	Model 1a + *co-parent’s exposure*	N/A
Model 2a: standard—unadjusted for co-parent	Controls for key potential confounders	Child’s sex, child’s age at outcome measurement^*^, parent-of-interest’s ethnicity, *age at conception/delivery, parity*^†^*, education, occupation, smoking/alcohol/caffeine*^§^	Child’s sex, child’s age at outcome measurement^*^, parent-of-interest’s ethnicity, *age at conception/delivery, parity*^†^	Child’s sex, child’s age at outcome measurement^*^, parent-of-interest’s age at DNA collection, 10 PCs
Model 2b: standard—adjusted for co-parent	Additionally controls for the co-parent’s exposure	Model 2a + *co-parent’s exposure*	Model 2a + *co-parent’s exposure*	Model 2a + *co-parent’s GRS*
Model 3a: standard with time-specific exposure—unadjusted for co-parent	Model 3 is only run when the exposure was measured during or post-pregnancy at repeat timepoints (see S3 File). It controls for correlation with previous timepoints to achieve a more time-specific estimate	Child’s sex, child’s age at outcome measurement^*^, parent-of-interest’s ethnicity, age, parity^†^, smoking/alcohol/caffeine^§^, *the main exposure in previous timepoints*	N/A	N/A
Model 3b: standard with time-specific exposure—adjusted for co-parent	Additionally controls for the co-parent’s exposure	Model 3a + *co-parent’s exposure*	N/A	N/A
Model 4a: standard—unadjusted for co-parent and adjusted for potential mediators	Explores the direct effect of the exposure on the outcome by controlling for factors that (most likely) occur after the exposure and could sit on the causal pathway to the outcome	Child’s sex, child’s age at outcome measurement^*^, parent-of-interest’s ethnicity, age, parity^†^, smoking/alcohol/caffeine^§^, *gestational age at delivery, birthweight, child’s passive smoking, child’s caffeine consumption*	Child’s sex, child’s age at outcome measurement ^*^, parent-of-interest’s ethnicity, age, parity^†^, *gestational age at delivery, birthweight, child’s passive smoking, child’s caffeine consumption*	Child’s sex, child’s age at outcome measurement^*^, parent-of-interest’s age at DNA collection, 10 PCs, *child’s GRS*
Model 4b: standard—adjusted for co-parent and adjusted for potential mediators	Additionally, controls for the co-parent’s exposure	Model 4a + *co-parent’s exposure*	Model 4a + *co-parent’s exposure*	Model 4a + *co-parent’s GRS*

*Child’s age was only included where there was variation in age within an outcome time point.

†Parity was only included where the parent-of-interest was the mother.

§The two health behaviours that were not the main exposure were included; PCs: ancestry-informed principal components derived from genetic data. Italic text denotes variables that are new compared to the previous model.

The minimally-adjusted models (models 1a/1b) included parent-of-interest’s ethnicity because this was a particularly important source of variation in health behaviours, especially for Born in Bradford, which had high ethnic heterogeneity. We considered the standard set of confounder-adjusted models (models 2a/2b and 3a/3b) to be our main models.

The mediator-adjusted models 4a and 4b were run as a sensitivity analysis and results should be interpreted with caution because adjusting for mediators on the causal pathway can introduce collider bias [24]. Mediators were included only where the outcome was measured *after* the potential mediator (e.g., child’s exposure to passive smoke postnatally was not included in models where the outcome was birthweight).

To allow exploration of sex-specific effects (which are supported by some DOHaD studies [25]), all models were additionally run stratified by sex of the child. We did not compare results between the sexes and the results of these models are not discussed further in this paper, but full results are available for others to explore and download via EPoCH Explorer.

### Processing of cohort results

To uphold the privacy of participants, we removed results from analyses where the total sample size was <20, or any binary exposure or outcome group had *n* < 5. We also removed results with an implausible effect estimate, which likely arose due to the model failing to converge (see S3 File).

### Meta-analysis

Where more than one cohort contributed results to an exposure/outcome combination, we estimated the weighted average effect across cohorts by inverse-variance weighted meta-analysis using the metafor package in R. Fixed-effects meta-analysis was used, because this is an appropriate method when there are a small number of studies [26]. Heterogeneity was assessed using I^2^ and the heterogeneity *P*-value, and visually via forest plots.

### Processing of final results

We generated results from 594,326 analyses, which included a mix of regression results from single cohorts (where only one cohort had the required data for that analysis) and meta-analysis results (where two or more cohorts had the required data). *P*-values for each model were then corrected for multiple testing using a false discovery rate (FDR) of 5%. To aid visual comparisons between binary and continuous outcome results, we converted the log odds ratios for binary outcomes to Cohen’s *D* [27] (standardised mean difference) which is a unitless measure of effect (equation provided in S3 File). Effect estimates are presented in their raw format (i.e., not as Cohen’s *D*) in the full set of results. To compare models in this paper succinctly, we categorised results by applying commonly used thresholds: FDR-adjusted *P* < 0.05 to define “statistical significance”, and *D* > 0.2 to define anything other than a “small” effect.

### Causal inference approaches and triangulation

Our analyses allowed us to apply multiple causal inference approaches to strengthen causal interpretation of associations. In the analyses presented in this paper, the causal hypothesis was that there is a direct effect of the parental exposure *during pregnancy*. The approaches we applied were: (1) multivariable regression adjusting for potential confounders; (2) applying the principles of MR by instrumenting parental health behaviours with GRS [11]; (3) employing co-parent’s exposure as a negative control to account for shared familial and environmental confounding [13]; (4) using postnatal exposure as a negative control for pregnancy exposure [13]; (5) exploring dose-response relationships where possible [12]. Detailed methodology, including the assumptions and source of bias for each of these approaches, is outlined in S3 File. Causal evidence was evaluated based on predefined criteria (Table 2). Triangulation [7] was conducted for each exposure-outcome combination by summarising the number of analyses that provided causal evidence. When the mother was the parent-of-interest, strongest causal evidence was defined as support from five agreeing lines of evidence. When the partner was the parent-of-interest, we excluded the parental comparison negative control from this count—since the underlying assumptions are less likely to hold in this context (see discussion in S3 File)—and therefore defined strongest causal evidence as support from four agreeing lines of evidence.

**Table 2:** Causal inference approaches and pragmatic criteria for evidence suggestive of a causal effect during pregnancy.

Method	Exposure or exposure comparisons	Model or model comparisons	Pragmatic criteria for evidence suggestive of a causal effect
Multivariable regression (MVR)	Health behaviour^*^ ever in pregnancy	Model 2b	FDR-adjusted *P* < 0.05
Mendelian randomisation (MR) informed approach using parental genetic risk scores (GRS)	Genetic risk score for health behaviour^†^	Model 2a (see S3 File for justification)	*P* < 0.05 and directionally consistent with the equivalent observational effect identified in the MVR analysis
Parental comparison negative control *(assumptions are more likely to hold when the mother is the parent-of-interest)*	Health behaviour ever in pregnancy	Model 2b for parent-of-interest vs. equivalent model 2b for co-parent	*P* < 0.05 and directionally consistent after adjustment for the co-parent’s exposure, with a greater absolute effect estimate for the parent-of-interest than the co-parent
Postnatal exposure negative control	Health behaviour ever in pregnancy vs. health behaviour in the first two postnatal years	Model 2b for pregnancy exposure vs. model 3b for postnatal exposure	A greater absolute effect estimate for the exposure during pregnancy than the exposure postnatally (with the postnatal estimate having been adjusted for the exposure during pregnancy)
Dose response	Heavy levels of the health behaviour during pregnancy vs. light levels of the health behaviour during pregnancy	Model 2b	Any exposure time point during pregnancy (i.e., first, second, or third trimester) with an effect estimate that is greater for heavy compared to light doses of the exposure

*We select the ‘main’ health behaviour definition for these analyses, i.e., active smoking, any alcohol consumption, and caffeine consumption from any source (rather than passive smoking, binge drinking, or caffeine from tea/coffee/cola).

†For smoking, which has multiple GRSs, we use the GRS for smoking initiation because it has the strongest association with the phenotype.

### Comparison to low SEP

For each outcome, we compared the estimated effect of each health behaviour in pregnancy (ever versus never) to the estimated effect of either measure of low SEP (i.e., based on education or occupation; lowest group versus higher groups). For these analyses, we used model 2a (i.e., the standard adjustment set) to ensure that the health behaviour model was adjusted for SEP while maximising power and minimising selection by not adjusting for the co-parent’s health behaviour. If the association between low SEP and the outcome is similar to, or greater than, the association between the health behaviour and the outcome, then the effect of the health behaviour might reflect residual confounding by SEP. In this case, the health behaviour may not be a specific or strong causal factor but rather part of a broader pattern of disadvantage associated with low SEP.

### EPoCH Explorer

To share, visualise, and draw inferences from our results, we developed EPoCH Explorer, an RShiny web app, which can be accessed at https://gcsharp.shinyapps.io/EPoCH/. Via EPoCH Explorer, users can view cohort data summaries and generate data visualisations of regression and meta-analysis summary statistics. Verbal interpretations of results are provided, as well as a causal inference report that presents all information needed to triangulate and contextualise the causal evidence for any exposure specified by the user. EPoCH Explorer also allows users to download the full set of summary statistics for EPoCH.

### Open science and reproducibility

The general analysis plan was outlined prospectively in the funding proposal, which is included, along with a list of changes and their rationales, in S4 File. It has been developed iteratively and checked (see S4 File) to improve reproducibility. This paper is written in accordance with the Strengthening Reporting of Observational Studies in Epidemiology (STROBE) guidelines for reporting of observational [28] (checklist in S5 File) and MR studies [29] (checklist in S6 File).

### Ethics

Each cohort obtained ethical approval and written participant consent for primary data collection. No separate ethical approval was sought for this secondary data study, but it was approved by the executive boards for ALSPAC (ref B3123), BIB, and MoBa (MCS data were accessed via the UK Data Service directly).

## Results

### Sample sizes

Without stratification by sex, sample sizes ranged from 35 to 232,139 (mean = 18,554) and varied according to the model, the availability of data across cohorts, and missing values for participants within cohorts. For our standard models 2a and 2b (unstratified by sex, adjusted for the standard set of covariates), the number of analyses involving more than one cohort was 8955/29146 (31%) for model 2a and 7760/28171 (28%) for model 2b. See Table A in S7 File for a detailed summary of the number of cohorts and sample sizes for each model. The maximum sample size decreased by up to 22% after adjustment for a standard set of confounders (i.e., from model 1–2), but only up to 3.1% after further adjustment for potential mediators (i.e., from model 2 to model 4). It is not possible to make a direct comparison with model 3 because this model was only run for health behaviours in the second trimester, third trimester, or postnatally, and total sample sizes were lower for these exposure variables.

### Sample characteristics

**Fig 1:**
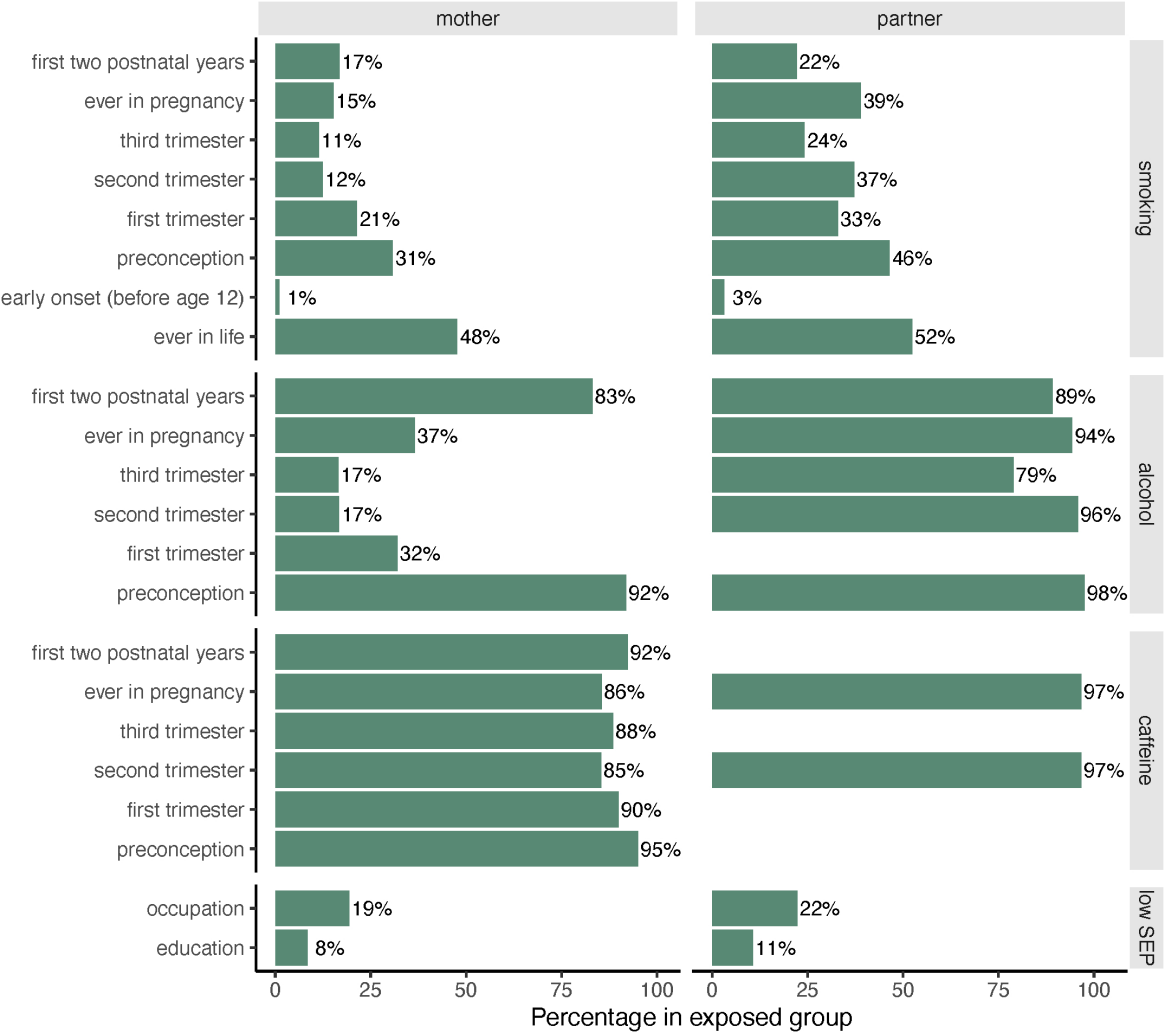
Summary of parental exposures in EPoCH. Percentages are the exposed sample pooled across all contributing cohorts, divided by the total non-missing sample pooled across those cohorts. SEP, socioeconomic position.

Fig 1 summarises the percentage of the sample (pooled across cohorts where multiple cohorts contributed) that were in the exposed group for each binary exposure variable. Mothers self-reported lower levels of smoking and alcohol consumption than partners did, particularly during pregnancy. For caffeine consumption, differences between parents and pregnancy status were less apparent. Less than 23% of parents were categorised in the lowest SEP groups. In all cohorts, low SEP was correlated with high smoking (and caffeine consumption except in MoBa). In all cohorts except BiB, low SEP was weakly correlated with low alcohol consumption (Fig A in S7 File). In general, cohorts had prioritised collecting data on mothers, so more variables were available (S2 File), and the data were more complete compared to data on partners. S8 File contains a detailed breakdown of sample characteristics by cohort, including the distribution and level of missingness for each variable.

### General trends

Across all models (i.e., models 1a to 4b across all health behaviours, GRS, SEP, and outcomes), effect estimates were generally small (absolute Cohen’s *D* < 0.2) and P-values large (FDR-adjusted *P* > 0.05). In our standard models, the percentage of results with *D* > 0.2 and FDR-*P* < 0.05 (i.e., strong statistical evidence of a larger-than-small associations) was 6% before adjustment for the co-parent’s exposure (model 2a), and 3% after (model 2b).

We did not find widespread evidence of much stronger effects in mothers than partners: after mutual adjustment (model 2b), 51% of analyses had a larger effect estimate for mothers than partners, and 52% had a smaller *p*-value for mothers than partners. The percentage of model 2b results with *D* > 0.2 and FDR-*P* < 0.05 was 7% for mothers, compared to 5% for partners.

**Fig 2:**
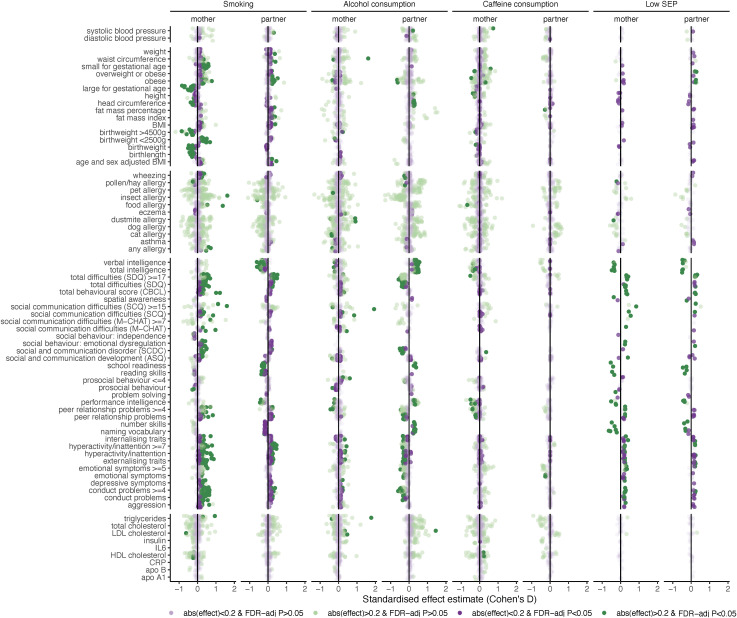
Summary of results by exposure and outcome class. Presented for the standard regression model with adjustment for co-parent’s exposure (model 2b). Dark green indicates the strongest evidence (FDR < 0.05) of a larger-than-small effect (*D* > 0.2). SEP, Socioeconomic Position; BMI, Body Mass Index; SDQ, Strengths and Difficulties Questionnaire; CBCL, Child Behavioural Checklist; M-CHAT, Modified Checklist for Autism in Toddlers; SCQ, Social Communication Questionnaire; SCDC, Skuse Social Communication Scores; ASQ, Ages and Stages Questionnaire; LDL, Low Density Lipoprotein; HDL, High Density Lipoprotein; IL6, Interleukin-6; IL7, Interleukin-7; Apo-A1, Apolipoprotein A1; Apo-B, Apolipoprotein B.

We observed some variation between exposure and outcome classes (Fig 2 and Fig B in S7 File). Across all outcomes, the strongest associations (*D* > 0.2 and FDR-*P* < 0.05) were seen with low SEP. In model 2b (standard adjustment with adjustment for the exposure in the co-parent), 15% of the results for low SEP had *D* > 0.2 and FDR-*P* < 0.05, compared to 6% for smoking, 3% for alcohol, and 0.4% for caffeine. Across all exposures, strongest associations were seen with psychosocial and cognitive outcomes (especially when the exposure was low SEP, smoking, or partner’s alcohol consumption) and body size and composition (especially when the exposure was smoking). For smoking, caffeine, and SEP, we saw a consistent pattern: a slightly higher proportion of strong associations for mothers compared to partners. In contrast, for alcohol, the proportion of strong associations was higher for partners. Notably, the direction of the partner association was often opposite to that seen for mothers (particularly for psychosocial outcomes), suggesting a potential protective effect of partner’s alcohol consumption during pregnancy. However, associations with the partner alcohol GRS (Fig F in S7 File; *F*-statistic for instrument strength = 10 (Fig B in S1 File)) were mostly null, indicating that this apparent protective effect could be explained by residual or unmeasured confounding. We found no clear patterns according to the timing of exposure or age at which outcomes were assessed (Figs C to E in S7 File).

### Triangulation of evidence for causal effects in pregnancy

**Fig 3:**
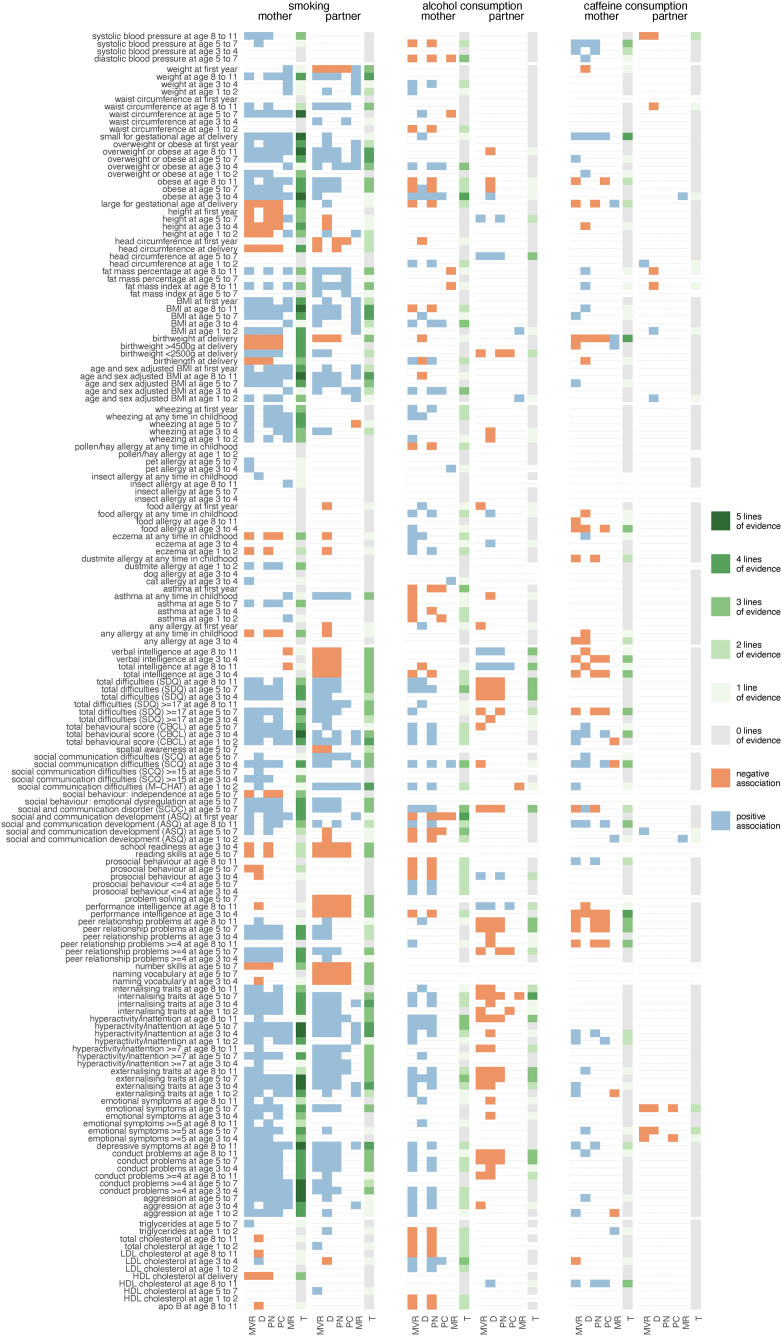
Triangulation of evidence for effects of parental health behaviours during pregnancy. MVR, multivariable regression (model 2b); PC, parental comparison negative control (model 2b); MR, mendelian randomisation-informed genetic risk score (model 2a); D, dose–response (model 2b); PN, postnatal comparison negative control (model 3b); T, triangulation summary indicating the number of lines of evidence across different analytical approaches that agree on the direction of association and have regression *P* < 0.05. BMI, Body Mass Index; SDQ, Strengths and Difficulties Questionnaire; CBCL, Child Behavioural Checklist; M-CHAT, Modified Checklist for Autism in Toddlers; SCQ, Social Communication Questionnaire; SCDC, Skuse Social Communication Scores; ASQ, Ages and Stages Questionnaire; LDL, Low Density Lipoprotein; HDL, High Density Lipoprotein; IL6, Interleukin-6; IL7, Interleukin-7; Apo-A1, Apolipoprotein A1; Apo-B, Apolipoprotein B.

In model 2b (standard adjustment with adjustment for the exposure in the co-parent), the number of analyses where the exposure was a health behaviour that occurred *during* pregnancy was 10,564 for mothers and 6,049 for partners. Of these, 1,099 (10%) and 585 (9%), respectively, had FDR-*P* < 0.05. Fig 3 illustrates triangulation of causal evidence for each of these associations. Note that a lack of evidence does not indicate evidence *against* a causal effect during pregnancy; it may reflect limited data availability or differences in assumptions across contexts. For most exposure-outcome combinations, the direction of association is consistent between the equivalent observational (MVR) and GRS (MR-informed) analyses. However, for caffeine consumption, this is not always the case, which could reflect residual confounding in the observational (MVR) estimates, or bias in the MR-informed results if the GRS is a “weak instrument”—that is, only weakly related to the reported exposure (see S1 File for *F*-statistics for instrument strength).

For mothers, we found five agreeing lines of evidence across analytical approaches for an effect of smoking during pregnancy on: small for gestational age (SGA) at delivery; waist circumference at age 5–7, BMI and overweight/obese at age 8–11; conduct problems, externalising traits, and hyperactivity/inattention at ages 3–4 and 5–7; aggression at age 5–7; total behavioural issues at age 3–4; and depressive symptoms at age 8–11 (full results and heterogeneity statistics presented in Table B in S7 File). It should be noted that, when adjusting for child’s and co-parent’s GRS (i.e., Model 4b) in the MR analysis (which can be an appropriate strategy to reduce genetic confounding and collider bias [30]—see S3 File for a discussion), *P*-values were >0.05 for the body size and composition outcomes, possibly due to low statistical power. The body size and composition outcomes were all more strongly associated with maternal smoking than with low SEP (i.e., model 2a MVR effect estimates were further from the null). However, the psychosocial outcomes were more strongly associated with low SEP than maternal smoking.

For partners, we found four agreeing lines of evidence for an effect of smoking during pregnancy on BMI at age 5–7, BMI, overweight/obese, and weight at age 8–11; internalising traits at age 1–2 and 5–7; total behavioural issues at age 1–2, and social communication difficulties at age 1–2. We also found four agreeing lines of evidence for an effect of partner’s alcohol consumption during pregnancy on internalising traits (full results and heterogeneity statistics presented in Table C in S7 File). Additional adjustment for child’s and co-parent’s GRS (Model 4b) resulted in P-values >0.05 for the body size and composition outcomes and the associations with alcohol consumption. All identified outcomes were more strongly associated with low SEP than with partner health behaviours. It should also be noted that, although the parental comparison negative control was not included in the triangulated evidence for partner effects (because the assumptions are less likely to hold), the maternal effect was generally further from the null than the partner effect for these exposure-outcome combinations. This pattern may indicate residual confounding by maternal exposure, or the presence of independent maternal and partner effects. The exceptions were smoking-social communication difficulties and alcohol-internalising traits, where findings instead suggest specific partner effects.

### Example of using EPoCH Explorer to triangulate causal evidence

**Fig 4:**
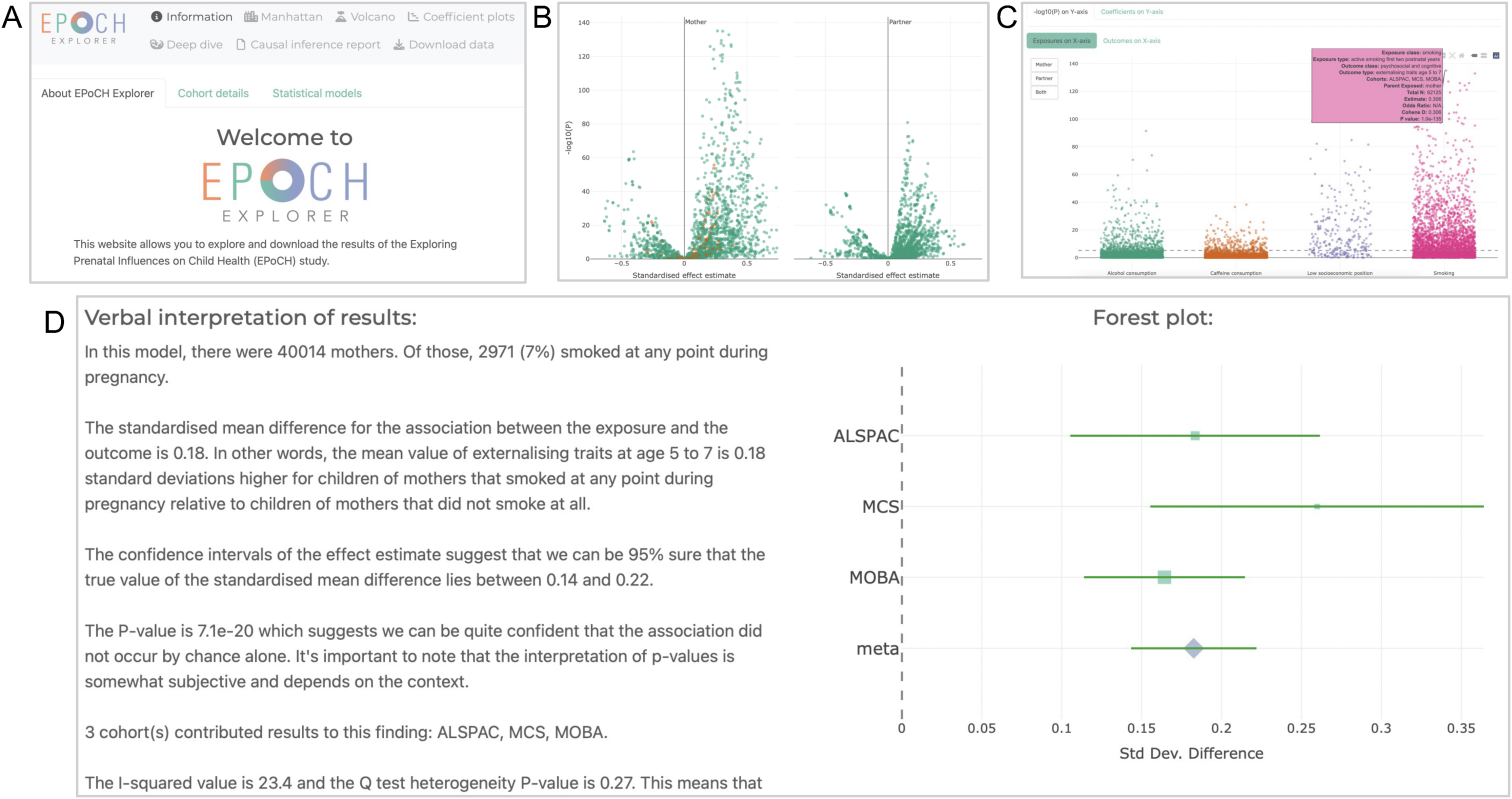
Screenshots from EPoCH Explorer. **(A)** The navigation bar and top of the welcome page. **(B)** An interactive volcano plot comparing maternal to partner estimates for smoking and psychosocial outcomes. **(C)** An interactive Manhattan plot showing the distribution of P-values by exposure class for all psychosocial outcomes. **(D)** A ‘Deep Dive’ summary of the results from model 2B, including a verbal interpretation of results and an interactive forest plot. EPoCH Explorer can be accessed at: https://gcsharp.shinyapps.io/EPoCH/.

Fig 4 shows screenshots from EPoCH Explorer to illustrate how a researcher might use the app to refine and assess evidence for (as an example) a hypothesis about prenatal influences on child psychosocial outcomes. To select an outcome of interest, the researcher could start by generating a Manhattan plot of the minimally adjusted (model 1a) results for psychosocial traits, which would reveal that the strongest associations are between maternal smoking during pregnancy and child externalising traits at age 5–7. To explore this hypothesis further, they could generate a causal inference report (example in S9 File), which would show that: (1) the MVR association persists after adjusting for potential confounders; (2) the direction of the estimate for the smoking initiation GRS aligns with the MVR estimate; (3) the maternal estimate is larger than the partner’s; (4) the estimate is larger for smoking during pregnancy than postnatally, (5) a dose-response relationship exists. These five lines of evidence support a causal effect of maternal smoking in pregnancy on externalising traits at age 5–7 years. The effect estimate for maternal smoking ever in pregnancy (model 2a *β* = 0.21 (95% confidence interval (CI) [0.18,0.25], *P* = 1.4 × 10^−31^) is smaller than the effect estimates for low SEP (education *β* = 0.30,95% CI [0.26, 0.34], *P* = 2 × 1 0^−46^, occupation *β* = 0.26, 95% CI [0.21, 0.31], *P* = 1.5 × 10^−23^), which suggests the effect may not be specific to smoking, but part of a broader pattern of disadvantage. The researcher could then perform a ‘Deep Dive’ to explore (for model 2b) cohort sample sizes, summary statistics, a forest plot, and a verbal interpretation of the findings. This shows that three cohorts (ALSPAC, MCS, MoBa) contributed to the meta-analysis, with a sample size of 40,014 (*n* smokers = 2,971; 7%), and low heterogeneity (*I*^2^ = 23.4; heterogeneity *P*-value = 0.27). The researcher could also use EPoCH Explorer to investigate the impact of stratifying by sex, or adjusting for mediators (i.e., comparing to model 4). Finally, the researcher could download all results to analyse offline and/or to compare to their own data.

## Discussion

In this largescale systematic exploration—of four types of exposure in both parents at up to nine timepoints, on 72 child outcomes at up to four ages—we did not find strong evidence for widespread or large magnitude effects of parental health behaviours on child health and wellbeing (although we note that a lack of evidence does not indicate evidence *against* a causal effect; it may reflect issues such as low power, measurement error, and missing data—see Limitations). We did find consistent evidence for parental smoking during pregnancy on a small number of offspring outcomes, including maternal smoking increasing small for gestational age, partner smoking increasing social communication difficulties, and both parents increasing childhood BMI, depressive symptoms, and behavioural issues. In general, we found stronger evidence of association with low SEP compared to health behaviours, suggesting that wider familial socioeconomic conditions may be a more important determinant of child health than specific parental health behaviours prenatally.

To our knowledge, few studies have directly compared the relative contributions of parental behaviours and socioeconomic conditions within a DOHaD framework, particularly at this scale. Our findings support calls for DOHaD research to broaden the focus from maternal (mostly intrauterine) exposures to paternal and wider societal influences [2,3,31–33]. This follows increasing evidence of paternal influences on child health, as outlined in recent systematic reviews in humans and/or animals [34–38]. These reviews noted the high risk of bias in the included studies, including inadequate adjustment for maternal exposures (and therefore assortative mating [39]). We have previously shown that, mirroring what we see for humans, there is a paucity of animal research on paternal effects in the DOHaD literature [2].

The widespread association we identified between low SEP and poorer offspring health outcomes agrees with the large body of research underscoring the significant impact of socioeconomic factors on child health, including adiposity and behavioural difficulties [40–42].

Our finding of higher partner alcohol consumption during pregnancy being associated with psychosocial and cognitive outcomes is in line with the well-established J-shape relationship between alcohol consumption and health, whereby light to moderate alcohol consumption is associated with better health outcomes due to socioeconomic confounding [43]. Therefore, the positive associations between partner alcohol and child psychosocial outcomes (in our fully adjusted models, i.e., after adjustment for SEP) are likely to be explained by residual confounding by SEP. We hypothesise that the maternal estimates are not affected in the same way because mothers reduce their alcohol consumption during pregnancy to a much larger degree than partners. Indeed, before pregnancy, the trends for partners and mothers were more similar (Fig 2 in S7 File).

Our evidence of effects of maternal smoking during pregnancy on small for gestational age agrees with a large body of literature, including studies that have applied approaches to infer causality [44–46]. The possible effect of maternal smoking on childhood BMI corroborates some but not all previous studies: despite strong evidence of association from multivariable regression analyses [10], studies that have used a parental negative control design [13] or Mendelian randomisation [47] have yielded null results. The association between maternal smoking in pregnancy and depressive symptoms in children is less well characterised, but a study using a parental negative control design found little evidence for a causal effect on depressive symptoms or internalising traits [48], and another found that a more thorough adjustment for parental psychosocial factors attenuated the association with internalising traits to the null [49]. Additionally, an MR study in UK Biobank [50] and a multicohort study using a negative control and cross-cohort design [51] have both found limited support for a causal effect on *adult* depression. Previous studies on maternal smoking in pregnancy and behavioural issues have shown mixed results. A systematic review [52] found that 13 out of 19 studies reported a positive association with ADHD, but six studies concluded the association was due to confounding. Large genetically informed studies, including a meta-analysis of over 1 million participants, provide no evidence for a causal association between maternal smoking during pregnancy and offspring ADHD [53]. Evidence from sibling-comparison, parental-comparison, and polygenic score analyses suggests that observed associations are largely explained by genetic and familial confounding, and that paternal smoking may not be a valid negative control for assessing intrauterine effects of maternal smoking on ADHD and related traits [54]. Finally, our finding of consistent evidence for an effect of partner smoking during pregnancy on social communication difficulties corroborates a previous study showing an association with autistic traits [55], but contrasts another showing no association [56]. Neither study applied approaches to infer causality. Differences between our results and those of previous studies could be explained by differences in definition, measurement, and timing of exposures/outcomes, adjusted covariates, statistical power, and populations studied.

*Strengths of the* EPoCH study include the novel, ambitious, and comprehensive approach to systematically explore potential causal effects of maternal and partner prenatal exposures and child outcomes at different timepoints. The use of prospective longitudinal population-based data minimises recall bias and enables associations to be examined over time. By harmonising data across cohorts, we were able to maximise comparability. For some analyses, the sample size was very large supporting precise estimates.

We focus our analyses on potentially modifiable parental health behaviours, and importantly, provide a useful, free, open resource for the research community (the EPoCH Explorer web app). The interactive data visualisations enable researchers to explore our results, and generate and assess evidence for their own hypotheses. It allows users to download the full set of results for use in their own analyses, thereby facilitating further meta-analysis and comparisons to other populations. The causal inference report and ‘Deep Dive’ verbal interpretation of results includes explanations that make EPoCH Explorer a valuable resource for students of epidemiology and others interested in understanding the data.

*The results should be interpreted in light of several limitations.* Despite our attempt to harmonise variables across studies, differences in availability of data meant that up to two-thirds of our analyses were conducted within a single cohort. Therefore, the sample size was relatively small for some analyses (range 35–256,387, mean = 17,326). The generalisability of our findings is also constrained by the low ethnic and socioeconomic diversity across the cohorts, except for BiB. In addition, all cohorts are based in high-income countries in Northern Europe, so it is unclear whether our findings apply to other populations and settings.

Some of the variation in sample size across analyses arises due to missing data within collected variables. Due to the number and complexity of our analyses, we chose to use a complete-case approach and did not employ multiple imputation techniques. The exclusion of participants with incomplete data may have introduced bias, particularly if the data are not missing at random; in such cases, multiple imputation would also be unlikely to fully address this bias. This limitation may affect the representativeness of the sample, reduce precision, and potentially induce collider bias if missingness is related to both exposures and outcomes. Additionally, as with all self-report data, there may be recall and measurement errors that could affect validity.

The inclusion of non-biological partners can be considered a strength (e.g., our approach is inclusive of same-sex parents, maximises sample size, and is beneficial for controlling for shared household confounding), but it may not fully describe the partner effect if the mechanism is hypothesised to operate via paternal genetics, sperm (e.g., de novo genetic mutations, sperm function, epigenetics, cytoplasmic factors) or other constituents of semen. However, we were limited by the lack of information about paternity status for some cohorts. The inclusion of non-biological partners could mean that mothers are more genetically related to study children than partners are. Although this might bias partner estimates (in either direction depending on exposure/outcome), sensitivity analyses have shown that the bias is minimal, even with implausibly high simulated rates of non-paternity [57]. Similarly, higher rates of missing data for partners relative to mothers have the potential to introduce bias, particularly as we have previously shown that those partners who are recruited tend to be of a higher SEP than those who are not [58]. However, our previous simulation study provides confidence that any resulting selection bias on the partner or partner-adjusted maternal estimate was likely to be small [58]. It should also be noted that for our GRS analyses (where biological paternity is central to the assumptions), all included partners *were* the biological fathers, as confirmed by the genetics data.

EPoCH focussed on outcomes in childhood (up to age 11), and we did not examine outcomes later in life, although we acknowledge that some outcomes may emerge later. This could be a focus of future large-scale systematic explorations. Additionally, we did not examine miscarriage or stillbirth. Given that our exposures could increase risk of these outcomes, our analyses may be affected by live-birth bias [59], a type of selection bias induced by restricting to live births. This is a potential limitation of many birth and child cohort studies. Fetal loss is difficult to study in ALSPAC or BiB, where the low number of cases result in insufficient statistical power, and impossible to study in MCS, because only families with a child around 9 months old were recruited. In addition, there are many other potential confounders, modifiers, and mediators of the relationships we studied, but we chose to focus on those that we felt would be most important and/or had been measured well and consistently between cohorts. Future research could explore the role of factors such as breastfeeding, housing conditions, early life infection, and parental psychological stress.

Finally, some GRSs were only weakly associated with the behaviour they were intended to represent (see S1 File for *F*-statistics). This means that the GRSs explained only a small proportion of the variance in the behaviour, which is likely to reduce the precision of the GRS-outcome estimates and limit their informativeness within the triangulation framework. We derived our GRSs using genome-wide significant variants identified in previous GWAS to help ensure that they specifically reflected the behaviours of interest; however, the strength of these scores depends on the sample size and power of the original GWAS. For smoking, alcohol, and caffeine consumption, we used the largest GWASs available at the time to maximise power. Including a wider set of genetic variants may capture more of the variation in these behaviours, but could reduce specificity and increase the risk of capturing pleiotropic pathways. Future research could explore alternative GRS construction approaches and conduct additional sensitivity analyses to assess the robustness of genetically informed results [60].

Our findings suggest that there is no strong scientific rationale to focus exclusively on maternal influences. Future DOHaD research should therefore avoid oversimplifying evidence on maternal pregnancy effects within a complex, socially patterned system, and instead broaden their scope to study data on partners, postnatal exposures, and wider social determinants of health. Partners are often overlooked in current preconception care policies and little guidance or supportive policies are available for them, or targeted at both parents together [61]. Behaviours such as smoking, caffeine, and alcohol consumption are socially patterned and often occur alongside other factors that may elevate the risk of poor health and psychosocial wellbeing in children. Interventions to address social inequalities (e.g., improving access to healthcare and ensuring good quality housing) are expected to improve childhood health outcomes [62–64].

In conclusion, we found limited evidence for large effects of parental prenatal health behaviours on childhood health and wellbeing, and stronger evidence of association with low SEP. This suggests that wider familial socioeconomic conditions may be a more important determinant of child health than specific parental health behaviours prenatally.

Our findings highlight the need for better data and more research into paternal/partner and wider societal or structural determinants of health.

## Supporting information

S1 File Detailed information about the contributing cohorts and variable definitions, including genetic risk scores. (DOCX)

S2 File Summary of availability of exposure and outcome data across each cohort. (DOCX)

S3 File Detailed information about statistical analyses. (DOCX)

S4 File Evidence of open science and reproducibility practices, including the original protocol and a list of changes to it and code and data availability. (DOCX)

S5 File STROBE guidelines for observational studies. Vandenbroucke JP, von Elm E, Altman DG, Gøtzsche PC, Mulrow CD, Pocock SJ, et al. Strengthening the Reporting of Observational Studies in Epidemiology (STROBE): explanation and elaboration. PLoS Med. 2007;4: e297. https://doi.org/10.1371/journal.pmed.0040297. This checklist is licensed under the Creative Commons Attribution 4.0 International License (CC BY 4.0; https://creativecommons.org/licenses/by/4.0/).
(DOCX)

S6 File STROBE guidelines for Mendelian randomisation studies. Skrivankova VW, Richmond RC, Woolf BAR, Davies NM, Swanson SA, VanderWeele TJ, et al. Strengthening the reporting of observational studies in epidemiology using mendelian randomisation (STROBE-MR): explanation and elaboration. BMJ. 2021; n2233. https://doi.org/10.1136/bmj.n2233. This checklist is licensed under the Creative Commons Attribution 4.0 International License (CC BY 4.0; https://creativecommons.org/licenses/by/4.0/).
(DOCX)

S7 File Additional results. (DOCX)

S8 File Description of the distribution of each variable in each cohort. (XLSX)

S9 File Examples of functionality of EPoCH Explorer. (DOCX)

## Abbreviations

ALSPAC: Avon Longitudinal Study of Parents and Children
BiB: Born in Bradford
BMI: body mass index
CI: confidence interval
DAGs: directed acyclic graphs
DOHaD: Developmental Origins of Health and Disease
EPoCH: Exploring Prenatal influences on Childhood Health
FDR: false discovery rate
GRS: genetic risk scores
GWAS: genome-wide association studies
MCS: Millennium Cohort Study
MoBa: Mother, Father, and Child Cohort Study
MR: mendelian randomisation
SGA: small for gestational age
STROBE: Strengthening Reporting of Observational Studies in Epidemiology
