# Supplementary material for: Exploring parental prenatal influences on child health: A multicohort study and data visualisation tool
Source: PLoS Med. 2026 Jul 23;23(7):e1005153. doi: 10.1371/journal.pmed.1005153 (PMC13395330; doi:10.1371/journal.pmed.1005153)
Supplement: S2 File — (DOCX) [file pmed.1005153.s002.docx]

**Data availability**

These figures show a summary of data available on exposures (**Fig A**) and outcomes (**Fig B**) across all four cohorts.

**Fig A.** Availability of data on exposures (light grey, i.e. unfilled squares, mean the data are either not available for any cohort, or the category is not applicable, for example, GRS in the third trimester).

*ALSPAC: Avon Longitudinal Study of Parents and Children; BiB: Born in Bradford; MCS: Millennium Cohort Study; MoBa: Norwegian Mother Father and Child Cohort Study. GRS: Genetic Risk Score; SEP: Socioeconomic Position.*

**Fig B.** Availability of data on outcomes (light grey, i.e. unfilled squares, mean the data are either not available for any cohort, or the category is not applicable, for example, birthweight at age 8 to 11).

*ALSPAC: Avon Longitudinal Study of Parents and Children; BiB: Born in Bradford; MCS: Millennium Cohort Study; MoBa: Norwegian Mother Father and Child Cohort Study. BMI: Body Mass Index; SDQ: Strengths and Difficulties Questionnaire; CBCL: Child Behavioural Checklist; M-CHAT: Modified Checklist for Autism in Toddlers; SCQ: Social Communication Questionnaire; SCDC: Skuse Social Communication Scores; ASQ: Ages and Stages Questionnaire; LDL: Low Density Lipoprotein ; HDL: High Density Lipoprotein; IL6: Interleukin-6; IL7: Interleukin-7; Apo-A1: Apolipoprotein A1; Apo-B: Apolipoprotein B.*
