# Supplementary material for: Exploring parental prenatal influences on child health: A multicohort study and data visualisation tool
Source: PLoS Med. 2026 Jul 23;23(7):e1005153. doi: 10.1371/journal.pmed.1005153 (PMC13395330; doi:10.1371/journal.pmed.1005153)
Supplement: S4 File — (DOCX) [file pmed.1005153.s004.docx]

Table of Contents

[Protocol and changes to it 1](#_Toc232429106)

[A. Original protocol 1](#_Toc232429107)

[B. Changes to the above protocol 5](#_Toc232429108)

[Reproducibility of code 6](#_Toc232429109)

[Data availability 6](#_Toc232429110)

# Protocol and changes to it

Below is the research plan submitted to the Medical Research Council in 2018 for what became the Exploring Prenatal influences on Childhood Health (EPoCH) study. It describes the project and analysis plan as it was originally conceived.

## Original protocol

Research questions

The overarching aim of this project is to improve childhood health by producing evidence on which to base effective advice and interventions on maternal and paternal health behaviours in the prenatal period. Specific research questions to be addressed are:

- Which maternal and paternal health behaviours in the prenatal period are associated with offspring health and social outcomes in childhood?
- Which of these associations are causal?
- Do maternal and paternal causal exposures influence offspring outcomes via independent, direct, indirect and/or interacting effects?

Addressing these research questions via the objectives described below will help identify the most appropriate prenatal targets for interventions to improve offspring childhood outcomes.

Variables of interest and work plan

This research will focus on parental health behaviours that can be considered potentially “modifiable” by intervention, occurring either during pregnancy or pre-conception (Table 1). Outcomes span three domains of child development occurring between birth and the end of childhood (up to around age 11), which minimises the influence of offspring own lifestyle behaviours for exposures like smoking and alcohol. Analyses will make use of existing data from multiple cohorts (Table 2) to maximise statistical power, replicability and the generalisability of findings across populations. Using existing data also means the project represents excellent value for money.

Statistical analysis plans will be drafted a priori for each cohort and harmonised definitions of exposures and outcomes will be devised, considering timing, dosage, scale, etc. For each aim, researchers employed on the grant will conduct analyses in each cohort individually (spending time at the appropriate institution housing the cohort where necessary), followed by meta-analysis. Two researchers will contribute to this work (the PI and one full time post-doc for the duration of the grant).

Table 1. Variables to be studied.

Objective 1: identifying parental prenatal exposures associated with offspring outcomes

Systematic reviews of the current literature have highlighted that observational evidence (regardless of causality) linking prenatal parental risk factors and offspring outcomes is often inconclusive and/or suffers from low power. This is likely to be particularly true of research on paternal exposures.

Objective 1.1: Multivariable regression analyses. Regression models will consider different combinations of maternal/paternal prenatal exposures and offspring outcomes from Table 1, with adjustment for appropriate confounders. For example, a model assessing associations between paternal prenatal alcohol and offspring IQ could adjust for maternal alcohol, maternal/paternal smoking, paternal/maternal BMI, paternal/maternal age, parity, ethnicity and socioeconomic position. To reduce biases associated with attrition, missing data will be imputed (see Reproducibility and Statistical Annex), and results will be compared to those from a complete case analysis.

Table 2. Data availability within the birth cohorts to be studied. [ALSPAC: Avon Longitudinal Study of Parents and Children; BiB, Born in Bradford; CC, Cleft Collective; MoBa, Norwegian Mother and Children Cohort Study; GenR, Generation R; DNBC, Danish National Birth Cohort Study] * CC recruitment ongoing (expected number by 09/2019).

Objective 2: inferring causality

Multiple methods will be used to infer whether associations between parental prenatal exposures and offspring outcomes represent causal relationships or are more appropriately explained by unknown or residual confounding factors. This will help identify exposures that might be modified to improve offspring outcomes.

Objective 2.1: Mendelian randomization (MR). MR is an epidemiological approach, pioneered by collaborators from the Integrative Epidemiology Unit (IEU), that uses genetic variants strongly associated with an exposure as a ‘proxy’ for that exposure in statistical models. MR can be used to infer whether observed associations are causal because genetic variants are (largely) inherited from parents at random and therefore likely to be independent of many potential confounding factors. MR also mitigates issues with self-reporting/recall bias and missing questionnaire data. Using a hypothesis-free “pheWAS” approach, we will apply MR to assess associations between genetic instruments (either individual SNPs or polygenic risk scores) and multiple offspring outcomes. Genotype data is available for most of the cohorts in Table 2. Genetic instruments (identified through Genome Wide Association Studies, GWAS) are available for smoking, alcohol consumption, caffeine, physical activity and some dietary constituents.

Objective 2.2: Parental-comparison negative-control design. This design compares the estimated effects of a maternal and paternal exposure to infer whether the maternal exposure is likely to act via a causal intrauterine mechanism (32). For example, if the effect estimates from separate regression models of childhood IQ on paternal and maternal alcohol intake were similar, this would suggest that the maternal alcohol-IQ association was not due to a causal intrauterine mechanism, but more likely due to confounding by shared parent-child genetics or environmental factors. This approach can be used even in the presence of a true paternal effect, because we would not expect the effects from both parents to be identical.

Objective 2.3: Discordant sibling design. This design compares the mean or risk of an outcome in siblings discordant for a prenatal exposure. For example, comparing mean IQ of children with fathers who drink alcohol to mean IQ of their siblings born after the fathers had given up. Since familial background will generally be similar for siblings, the design attempts to circumvent issues with confounding by shared familial environment and genetics. A variation of the discordant sibling design compares outcomes in half-siblings who share either a mother (maternal-half-siblings) or a father (paternal-half-siblings). A higher rate of low IQ in maternal half-siblings compared to paternal half-siblings would suggest that low IQ is largely determined by maternal factors (including the intrauterine environment), but the opposite observation would suggest that low IQ is largely determined by paternal factors. All cohorts in Table 2 recruited siblings.

Objective 2.4: Triangulation. Each of the methods described above (multivariable regression, MR, negative-control-comparison, sibling-comparison) has different sources of bias, but by integrating results from several different approaches, we can exploit these differences to draw qualitative conclusions about causal effects. For example, strongest evidence for a causal effect of paternal alcohol intake on IQ would be provided if findings generated using each method all suggest a causal effect, despite differing biases.

Objective 3: Refining the causal pathway to infer mediating/interacting maternal and paternal effects

Where associations between maternal and/or paternal prenatal exposures and offspring health outcomes appear to be causal, several methods will be applied to assess mediation and interaction between both parent’s exposure. When assessing mediation, we are interested in whether the maternal/paternal exposure has a direct effect on offspring health, or whether its effect is (partially) mediated by the other parent’s exposure. When assessing interaction, we are interested in whether the maternal and paternal exposure interact in their effects on offspring health, and how much of their effects are due to this interaction. These investigations will help elucidate whether interventions to improve health would be best targeted at mothers, fathers or both parents.

Objective 3.1: Conventional mediation analysis. The traditional approach involves comparing two regression models: one that adjusts for the proposed mediator and one that does not. An example in this project would be comparing models assessing the effect of a paternal alcohol intake on IQ with or without adjustment for maternal alcohol intake. The proportion of the total effect attributed to the mediator (maternal alcohol) is the ratio of the unadjusted to the adjusted relative risks. This is a crude approach that can introduce bias (e.g. collider bias), but it is included for comparison with more refined approaches.

Objective 3.2: Conventional interaction analysis. Two-way interaction effects will be assessed using regression models adjusted for an interaction term that either multiplies or sums the effect of the maternal and paternal exposure. Although multiplicative models are more commonly used in epidemiology, additive interaction may be of greater interest in disease/poor outcome prevention, and may exist even in the absence of multiplicative interaction.

Objective 3.3: Mediation analysis using network MR. Using genetic instrumental variables for an exposure (e.g. paternal alcohol intake) and a mediator (e.g. maternal alcohol intake), we will use an extension of the MR approach to estimate the direct and indirect effects on an outcome (e.g. IQ). This approach, called network MR, can also assess the direct effect of the exposure on the mediator (e.g. paternal on maternal alcohol).

Objective 3.4: Integrated mediation and interaction analysis based on counterfactual theory. We will use a counterfactual framework to address some of the limitations of conventional mediation analysis. The key advantages of counterfactual models are that they can estimate mediation parameters in the presence of exposure-mediator interactions and adjust for measured intermediate confounders. This allows assessment of what proportion of the effect is due to 1) just mediation, 2) just interaction, 3) both, or 4) neither.

Objective 3.5: The structured life course approach. This approach estimates statistical models that correspond to alternative hypotheses to explain a causal pathway, for example, a “critical period” model might consider periconceptional paternal alcohol intake as the main influence on IQ, but the equivalent “accumulation of risk” model would consider the accumulation of exposure to paternal alcohol throughout pregnancy, and the influence of maternal alcohol. Models are then compared (using a lasso approach) to select the hypothesis that explains the most amount of variance in the outcome, thereby helping to refine the causal pathway and infer mediating/interacting effects.

Objective 3.6: Triangulation. Conventional approaches, MR, counterfactual theory and the life course approach all have different sources and potentially directions of bias, that can be used to strengthen evidence for mediating/interacting effects when multiple methods are triangulated.

Reproducibility & statistical annex

The reliability and robustness of different aspects of the methodology and design will be ensured as follows:

- Multivariable regression. The distribution of variables will be plotted and considered when choosing the most appropriate regression method. Directed Acyclic Graphs (DAGs) will be used to help select confounders.
- Mendelian randomization. In addition to testing the main MR assumptions (e.g. checking for no association between the genetic instrument and confounders), recent guidelines for conducting intergenerational MR published by collaborator Lawlor will be followed. These include: demonstrating a robust association between genetic instrument and exposure in the prenatal period; adjusting for offspring genotype; and instrumenting with only maternal/paternal non-transmitted variants.
- Parental comparison negative control design. Compared to maternal data, paternal data may have a higher degree of measurement error (e.g. due to maternal-report rather than self-report) and missingness, which would bias paternal estimates towards the null and therefore inflate any difference between the maternal and paternal effect. High rates of non-paternity (whereby mothers are more genetically related to their children than the fathers are) would have a similar biasing effect. Strategies that will be used to circumvent these issues include: using objective predictors of exposures based on epigenetic biomarkers (derived from parental methylation) and testing the robustness of estimates to adjustments for simulated levels of non-paternity.
- Discordant sibling design. Familial environments can change between sibling births and introduce non-shared confounders that could bias estimates. This will be checked by comparing the distribution of confounders between exposed and unexposed siblings.
- Mediation analysis. Strong assumptions about confounding and an absence of measurement error are unlikely to be met, but are mitigated by only conducting mediation analysis (Objective 3) when there is causal evidence from Objective 2.
- Issues applying to all analyses: Missing data will be multiply imputed using fully conditional specification (FCS), under a missing at random assumption. Imputation will be carried out separately for each group of models and will be congenial with the analysis model, including all exposures and outcomes, plus auxiliary variables as appropriate. Sensitivity analysis for missing not at random will be conducted. In addition, MR will be used to circumvent issues around missing exposure data and measurement error. Sensitivity analyses stratifying by sex will be carried out to test for sex-specific effects. P-values will be adjusted for multiple testing using the FDR method. All analytical code will be shared publicly to aid reproducibility. Triangulation of multiple methods is a strategy employed in this project specifically to improve the robustness of conclusions.
- Power: A two-sided multivariable linear regression using 10 independent variables would have 80% power (alpha=0.05) to detect a standardised effect size (Cohen’s d) ranging from very small (0.0001) to large (0.35), given a sample size ranging from 178,491 to 26, respectively. Therefore, with a maximum sample size of 236,621 families, this study is very well powered, even taking account of a reduction in sample size due to missing data. In an MR analysis (which requires larger sample sizes than conventional multivariable regression), a sample size of 16,806 (the maximum number of families with paternal genetic data), would give between 10% to 90% power to detect odds ratios ranging 1.1 to 1.5, respectively (assuming the genetic variants explain 3% of the variance in the exposure, as is the case for SNPs for BMI, and the prevalence of the outcome is 10%). For the same parameters, a sample size of 38,470 (the maximum number of families with maternal genetic data) would provide between 17 and 100% power.

## Changes to the above protocol

The general analysis plan above was included in the funding application (to the MRC, submitted 2018). The funded project began in July 2019 and followed the general analysis plan closely, with some changes to the design over the following 6 years.

Importantly, the key design changes were made BEFORE any models were run and the changes were NOT prompted by findings.

Key refinements:

- Included cohorts: We had originally planned to include three additional cohorts: Danish Birth Cohort (DNBC), Generation R, and the Cleft Collective. However, due to overlap with ongoing studies of specific exposure-outcome combinations, DNBC and Generation R were not able to contribute to our exploratory systematic approach. The Cleft Collective is a patient cohort study of children born with a cleft lip and/or palate and participants have consented for their data to be used ONLY in studies of direct relevance to orofacial clefts. Since none of the other cohorts had collected sufficient data on cleft, it was not possible to include cleft as an outcome in this multicohort paper, and we did not feel it would be ethical to include Cleft Collective data in the other (non-cleft related) analyses. On the other hand, we had not initially planned to include Millennium Cohort Study, but decided to do so after discovering the necessary data had been collected and were available free of charge.
- Exposures: We had originally planned to include physical activity and diet (fat and sugar intake) as parental exposures, but decided to omit these due to heterogeneity in the ways these variables had been measured in different cohorts. Although the original analysis plan included socioeconomic position (SEP) as a covariate, we later included measures of SEP as exposures in their own right to help us contextualise the results for parental health behaviours.
- Outcomes: We had originally planned to include congenital anomalies as an outcome (particularly cleft lip and palate – see above) but later removed this due to low availability of data and small numbers of cases in the population-based cohorts. On discovering that the data were available for other outcomes, we expanded the list to include serum biomarkers, blood pressure, and additional psychosocial measures.
- Imputation: We had originally planned to apply multiple imputation to address missing data, however, we decided that this would be too complicated given the number of exposures, outcomes, and models. We also considered that if data are not missing at random (as is likely to be the case), multiple imputation would not necessarily help reduce bias. We therefore ran complete-case analyses and discuss this as a potential limitation in the manuscript.
- Causal inference approaches: We originally planned to include discordant sibling analyses, but later decided not to run these due to low numbers of siblings in the included cohorts (it was only really a viable option in MoBa). We had not originally planned to include a postnatal negative control or explore dose relationships, but decided to add these because the data were available and their addition contributed to the triangulation approach.
- Mediation analyses: We had planned to explore mediating mechanisms using conventional and more refined approaches (objective 3). At the time of writing, these analyses have not been run and will perhaps be the focus of a future paper.

# Reproducibility of code

Code to prepare the data in each cohort was written (in R version 4.2.2 or higher) by one author (GCS or KEE) and checked by the other. KEE wrote and GCS checked the PLINK code for generating GRS. GCS wrote and KEE checked the analysis R code. Analyses were run multiple times, allowing issues to be identified and corrected. EPoCH Explorer was initially coded in RShiny by GCS, optimised by EH, and further developed by GCS. All R code is available on GitHub: <https://github.com/gcsharp/EPoCH_analysis> and archived in Zenodo <https://doi.org/10.5281/zenodo.19607446>.

# Data availability

Access to individual level data is governed by each cohort and researchers can apply to the cohorts for access (ALSPAC: <https://www.bristol.ac.uk/alspac/researchers/access/> ; BiB: <https://borninbradford.nhs.uk/our-data/how-to-access-data/> ; MCS: <https://cls.ucl.ac.uk/cls-studies/millennium-cohort-study/> ; MoBa: <https://www.fhi.no/en/ch/studies/moba/for-forskere-artikler/research-and-data-access/>). All data preparation and analysis code necessary to reproduce the results is available from GitHub <https://github.com/gcsharp/EPoCH_analysis> and archived in Zenodo <https://doi.org/10.5281/zenodo.19607446>. All summary statistics are available via EPoCH Explorer <https://gcsharp.shinyapps.io/EPoCH/> and archived on Zenodo <https://doi.org/10.5281/zenodo.20490585>. The RShiny code for EPoCH Explorer is also available on GitHub: <https://github.com/gcsharp/EPoCH_results_app> and archived on Zenodo https://doi.org/10.5281/zenodo.19607459.
