# Supplementary material for: Exploring parental prenatal influences on child health: A multicohort study and data visualisation tool
Source: PLoS Med. 2026 Jul 23;23(7):e1005153. doi: 10.1371/journal.pmed.1005153 (PMC13395330; doi:10.1371/journal.pmed.1005153)
Supplement: S5 File — Vandenbroucke JP, von Elm E, Altman DG, Gøtzsche PC, Mulrow CD, Pocock SJ, et al. Strengthening the Reporting of Observational Studies in Epidemiology (STROBE): explanation and elaboration. PLoS Med. 2007;4: e297. https://doi.org/10.1371/journal.pmed.0040297. This checklist is licensed under the Creative Commons Attribution 4.0 International License (CC BY 4.0; https://creativecommons.org/licenses/by/4.0/). (DOCX) [file pmed.1005153.s005.docx]

# Reporting guidelines: observational cohort.

This paper is written in accordance with the STROBE guidelines for reporting of observational cohort studies [1].

STROBE Statement—Checklist of items that should be included in reports of cohort studies

|  | Item No | Recommendation | Section/ paragraph/ file |
| --- | --- | --- | --- |
| Title and abstract | 1 | (a) Indicate the study’s design with a commonly used term in the title or the abstract | Title (“multicohort”) |
|  |  | (b) Provide in the abstract an informative and balanced summary of what was done and what was found | Abstract; Author Summary |
| Introduction | | | |
| Background/rationale | 2 | Explain the scientific background and rationale for the investigation being reported | Introduction |
| Objectives | 3 | State specific objectives, including any prespecified hypotheses | Introduction (paragraphs 3-5) |
| Methods | | | |
| Study design | 4 | Present key elements of study design early in the paper | Introduction (paragraphs 3 and 4) |
| Setting | 5 | Describe the setting, locations, and relevant dates, including periods of recruitment, exposure, follow-up, and data collection | Methods (cohorts) and File S1 |
| Participants | 6 | (a) Give the eligibility criteria, and the sources and methods of selection of participants. Describe methods of follow-up | Methods (participants) and File S1 |
|  |  | (b) For matched studies, give matching criteria and number of exposed and unexposed | N/A |
| Variables | 7 | Clearly define all outcomes, exposures, predictors, potential confounders, and effect modifiers. Give diagnostic criteria, if applicable | Methods (Parental exposures, Genetic risk scores for parental exposures, child outcomes and age stages, covariates) and File S1 |
| Data sources/ measurement | 8 | For each variable of interest, give sources of data and details of methods of assessment (measurement). Describe comparability of assessment methods if there is more than one group | File S1 |
| Bias | 9 | Describe any efforts to address potential sources of bias | Methods: causal inference methods and File S3 |
| Study size | 10 | Explain how the study size was arrived at | Methods: Sample and File S1 |
| Quantitative variables | 11 | Explain how quantitative variables were handled in the analyses. If applicable, describe which groupings were chosen and why | Methods: statistical analysis and File S1 |
| Statistical methods | 12 | (a) Describe all statistical methods, including those used to control for confounding | Methods: statistical analysis and File S3 |
|  |  | (b) Describe any methods used to examine subgroups and interactions | Methods: statistical analysis and File S3 |
|  |  | (c) Explain how missing data were addressed | Methods: statistical analysis and File S3 |
|  |  | (d) If applicable, explain how loss to follow-up was addressed | Methods: statistical analysis and File S3 |
|  |  | (e) Describe any sensitivity analyses | Methods: statistical analysis and File S3 |
| Results | | | |
| Participants | 13 | (a) Report numbers of individuals at each stage of study—eg numbers potentially eligible, examined for eligibility, confirmed eligible, included in the study, completing follow-up, and analysed | File S1 |
|  |  | (b) Give reasons for non-participation at each stage | File S1 |
|  |  | (c) Consider use of a flow diagram | File S1 |
| Descriptive data | 14 | (a) Give characteristics of study participants (eg demographic, clinical, social) and information on exposures and potential confounders | Results (Sample characteristics) and File S5 and S6 |
|  |  | (b) Indicate number of participants with missing data for each variable of interest | Results (sample sizes) and File S6 |
|  |  | (c) Summarise follow-up time (eg, average and total amount) | File S5 and S6 (age of child) |
| Outcome data | 15 | Report numbers of outcome events or summary measures over time | File S6 and EPoCH Explorer: Cohort information |
| Main results | 16 | (a) Give unadjusted estimates and, if applicable, confounder-adjusted estimates and their precision (eg, 95% confidence interval). Make clear which confounders were adjusted for and why they were included | Methods Table 1, Results (General trends, Triangulation, Example of using EPoCH Explorer), File S3, EPoCH Explorer |
|  |  | (b) Report category boundaries when continuous variables were categorized | File S1, File S3 |
|  |  | (c) If relevant, consider translating estimates of relative risk into absolute risk for a meaningful time period | Not done, but full results outline proportion of cases/controls in exposed/unexposed groups to provide extra context on risk – also described in EPoCH Explorer |
| Other analyses | 17 | Report other analyses done—eg analyses of subgroups and interactions, and sensitivity analyses | EPoCH Explorer, full results available |
| Discussion | | | |
| Key results | 18 | Summarise key results with reference to study objectives | Discussion (Summary of results) |
| Limitations | 19 | Discuss limitations of the study, taking into account sources of potential bias or imprecision. Discuss both direction and magnitude of any potential bias | Discussion (Limitations) |
| Interpretation | 20 | Give a cautious overall interpretation of results considering objectives, limitations, multiplicity of analyses, results from similar studies, and other relevant evidence | Discussion (Implications for future research and practice, Conclusion) |
| Generalisability | 21 | Discuss the generalisability (external validity) of the study results | Discussion (Strengths, Limitations) |
| Other Information | | | |
| Funding | 22 | Give the source of funding and the role of the funders for the present study and, if applicable, for the original study on which the present article is based | Funding and acknowledgements |

1. Vandenbroucke JP, von Elm E, Altman DG, Gøtzsche PC, Mulrow CD, Pocock SJ, et al. Strengthening the Reporting of Observational Studies in Epidemiology (STROBE): explanation and elaboration. PLoS Med. 2007;4: e297. doi:10.1371/journal.pmed.0040297
