# Supplementary material for: Exploring parental prenatal influences on child health: A multicohort study and data visualisation tool
Source: PLoS Med. 2026 Jul 23;23(7):e1005153. doi: 10.1371/journal.pmed.1005153 (PMC13395330; doi:10.1371/journal.pmed.1005153)
Supplement: S6 File — Skrivankova VW, Richmond RC, Woolf BAR, Davies NM, Swanson SA, VanderWeele TJ, et al. Strengthening the reporting of observational studies in epidemiology using mendelian randomisation (STROBE-MR): explanation and elaboration. BMJ. 2021; n2233. https://doi.org/10.1136/bmj.n2233. This checklist is licensed under the Creative Commons Attribution 4.0 International License (CC BY 4.0; https://creativecommons.org/licenses/by/4.0/). (DOCX) [file pmed.1005153.s006.docx]

# Reporting guidelines: Mendelian randomization studies

This paper is written in accordance with the STROBE guidelines for reporting of Mendelian Randomization studies [1].

STROBE Statement—Checklist of items that should be included in reports of Mendelian Randomization studies

|  | Item No | Recommendation | Section/ paragraph/ file |
| --- | --- | --- | --- |
| Title and abstract | 1 | Indicate mendelian randomisation (MR) as the study’s design in the title and/or the abstract if that is a main purpose of the study | Not included in the title because not the main purpose of the study |
| Introduction | | | |
| Background/rationale | 2 | Explain the scientific background and rationale for the reported study. What is the exposure? Is a potential causal association between exposure and outcome plausible? Justify why MR is a helpful method to address the study question. | Introduction, Supplementary File S3 |
| Objectives | 3 | State specific objectives clearly, including prespecified causal hypotheses (if any). State that MR is a method that, under specific assumptions, intends to estimate causal effects | Introduction, Supplementary File S3 |
| Methods | | | |
| Study design and data sources | 4 | Present key elements of the study design early in the article. Consider including a table listing sources of data for all phases of the study. For each data source contributing to the analysis, describe the following: | Introduction, Methods, Supplementary File S3 |
|  |  | Setting: Describe the study design and the underlying population, if possible. Describe the setting, locations, and relevant dates, including periods of recruitment, exposure, follow-up, and data collection, when available. | Methods (cohorts) and File S1 |
|  |  | Participants: Give the eligibility criteria, and the sources and methods of selection of participants. Report the sample size, and whether any power or sample size calculations were carried out prior to the main analysis | Methods (participants) and File S1 |
|  |  | Describe measurement, quality control, and selection of genetic variants | File S1 |
|  |  | For each exposure, outcome, and other relevant variables, describe methods of assessment and diagnostic criteria for diseases | File S1 |
|  |  | Provide details of ethics committee approval and participant informed consent, if relevant | Methods (Ethics) and File S1 |
| Assumptions | 5 | Explicitly state the three core instrumental variable assumptions for the main analysis (relevance, independence, and exclusion restriction), as well assumptions for any additional or sensitivity analysis | File S1 |
| Statistical methods: main analysis | 6 | Describe statistical methods and statistics used: | Methods: statistical analysis and File S3 |
|  |  | Describe how quantitative variables were handled in the analyses (that is, scale, units, model) | Methods: statistical analysis, File S1 and File S3 |
|  |  | Describe how genetic variants were handled in the analyses and, if applicable, how their weights were selected | Methods: statistical analysis, File S1 and File S3 |
|  |  | Describe the MR estimator (eg, two stage least squares, Wald ratio) and related statistics. Detail the included covariates and, in the case of two sample MR, whether the same covariate set was used for adjustment in the two samples | Methods: statistical analysis and File S3 |
|  |  | Explain how missing data were addressed | Methods: statistical analysis and File S3 |
|  |  | If applicable, indicate how multiple testing was addressed | Methods: statistical analysis and File S3 |
| Assessment of assumptions | 7 | Describe any methods or prior knowledge used to assess the assumptions or justify their validity | Methods: statistical analysis, File S1 and File S3 |
| Sensitivity analyses and additional analyses | 8 | Describe any sensitivity analyses or additional analyses performed (eg, comparison of effect estimates from different approaches, independent replication, bias analytic techniques, validation of instruments, simulations) | Methods: Triangulation, File S3 |
| Software and pre-registration | 9 | Name statistical software and package(s), including version and settings used | File S1 and S3 |
|  |  | State whether the study protocol and details were pre-registered (as well as when and where) | Methods and File S4 |
| Results | | | |
| Descriptive data | 10 | Report the numbers of individuals at each stage of included studies and reasons for exclusion. Consider use of a flow diagram | File S1 |
|  |  | Report summary statistics for phenotypic exposure(s), outcome(s), and other relevant variables (e.g. means, SDs, proportions) | Results (Sample Description), File S5 and File S6 |
|  |  | If the data sources include meta-analyses of previous studies, provide the  assessments of heterogeneity across these studies | N/A |
|  |  | For two-sample MR:  i. Provide justification of the similarity of the genetic variant-exposure associations between the exposure and outcome samples  ii. Provide information on the number of individuals who overlap between the  exposure and outcome studies | N/A |
| Main results | 11 | Report the associations between genetic variant and exposure, and between genetic variant and outcome, preferably on an interpretable scale | Genetic variant and exposure: File S1; Genetic variant and outcome: EPoCH Explorer/full results |
|  |  | Report MR estimates of the relationship between exposure and outcome, and the measures of uncertainty from the MR analysis, on an interpretable scale, such as odds ratio or relative risk per SD difference | EPoCH Explorer/full results (mean difference or odds ratio per SD difference) |
|  |  | If relevant, consider translating estimates of relative risk into absolute risk for a meaningful time period | N/A |
|  |  | Consider plots to visualize results (e.g. forest plot, scatterplot of associations between genetic variants and outcome versus between genetic variants and exposure) | File S1 (heatmap of F-statistics), Results (general trends), EPoCH Explorer |
| Assessment of assumptions | 12 | Report the assessment of the validity of the assumptions | File S1, Results (Triangulation) |
|  |  | Report any additional statistics (e.g., assessments of heterogeneity across genetic variants, such as *I2*, Q statistic or E-value) | N/A |
| Sensitivity analyses and additional analyses | 13 | Report any sensitivity analyses to assess the robustness of the main results to violations of the assumptions | N/A |
|  |  | Report results from other sensitivity analyses or additional analyses | Results (Triangulation), File S1, File S3 |
|  |  | Report any assessment of direction of causal relationship (e.g., bidirectional MR) | N/A |
|  |  | When relevant, report and compare with estimates from non-MR analyses | Results (Triangulation), File S1, File S3 |
|  |  | Consider additional plots to visualize results (e.g., leave-one-out analyses) | EPoCH Explorer |
| Discussion | | | |
| Key results | 14 | Summarise key results with reference to study objectives | Discussion (Summary of results) |
| Limitations | 15 | Discuss limitations of the study, taking into account the validity of the IV assumptions, other sources of potential bias, and imprecision. Discuss both direction and magnitude of any potential bias and any efforts to address them | Discussion (Limitations) |
| Interpretation | 16 | Meaning: Give a cautious overall interpretation of results in the context of their limitations and in comparison with other studies | Discussion (Implications for future research and practice, Conclusion) |
|  |  | Mechanism: Discuss underlying biological mechanisms that could drive a potential causal relationship between the investigated exposure and the outcome, and whether the gene-environment equivalence assumption is reasonable. Use causal language carefully, clarifying that IV estimates may provide causal effects only under certain assumptions | Discussion (Comparison of findings to previous literature, Strengths, Limitations, Implications) |
|  |  | Clinical relevance: Discuss whether the results have clinical or public policy relevance, and to what extent they inform effect sizes of possible interventions | Discussion (Implications) |
| Generalisability | 17 | Discuss the generalizability of the study results (a) to other populations, (b) across other exposure periods/timings, and (c) across other levels of exposure | Discussion (Strengths, Limitations) |
| Other Information | | | |
| Funding | 18 | Describe sources of funding and the role of funders in the present study and, if applicable, sources of funding for the databases and original study or studies on which the present study is based | Funding and acknowledgements, File S1 |
| Data and data sharing | 19 | Provide the data used to perform all analyses or report where and how the data can be accessed, and reference these sources in the article. Provide the statistical code needed to reproduce the results in the article, or report whether the code is publicly accessible and if so, where | Methods (Open science and reproducibility, Data availability), File S1, File S4 |
| Conflicts of Interest | 20 | All authors should declare all potential conflicts of interest | Author information |

1. Skrivankova VW, Richmond RC, Woolf BAR, Davies NM, Swanson SA, VanderWeele TJ, et al. Strengthening the reporting of observational studies in epidemiology using mendelian randomisation (STROBE-MR): explanation and elaboration. BMJ. 2021; n2233. doi:10.1136/bmj.n2233
