# Supplementary material for: Exploring parental prenatal influences on child health: A multicohort study and data visualisation tool
Source: PLoS Med. 2026 Jul 23;23(7):e1005153. doi: 10.1371/journal.pmed.1005153 (PMC13395330; doi:10.1371/journal.pmed.1005153)
Supplement: Attachment — Submitted filename: PlosMedicine_ResponseToReviewers.docx [file pmed.1005153.s012.docx]

**We thank the editors and reviews for their detailed comments on our work.**

**Our responses are provided below, with revised text included in red font.**

**Comments from the editor:**

We discussed some of the reviewer comments with our statistical reviewer. They mentioned that the missing data is a limitation, that should be highlighted (although they mention that it is briefly noted in the discussion).

- Response: As the statistical reviewer noted, we acknowledge missing data as a limitation in the Discussion, and we have now expanded this: “Some of the variation in sample size across analyses arises due to missing data within collected variables. Due to the number and complexity of our analyses, we chose to use a complete-case approach and did not employ multiple imputation techniques. The exclusion of participants with incomplete data may have introduced bias, particularly if the data are not missing at random; in such cases, multiple imputation would also be unlikely to fully address this bias. This limitation may affect the representativeness of the sample, reduce precision, and potentially induce collider bias if missingness is related to both exposures and outcomes.”

They also stated that the suggestion of Reviewer #4 to provide an indication of the level of missingness in the different cohort is reasonable.

- Response: Supplementary file 6 contains columns for the number and percentage missing for every variable (exposure, covariate, outcome) in each cohort. We have now highlighted this in the main text: “Supplementary File 6 contains a detailed breakdown of sample characteristics by cohort, including the distribution and level of missingness for each variable.”

Although we would encourage you to address all reviewer comments, I do add that our statistical reviewer did think that the D threshold is reasonable to use, and that additional analyses may not be necessary, as the online tool allows users to explore specific effects.

- Response: We thank the statistical reviewer for highlighting this point. We explore results using Cohen’s D because it provides a standardised effect estimate that enables us to compare effect sizes across different scales and types of outcome (core to our aim to provide a systematic exploration of our hypothesis), and the threshold selected is commonly used to indicate “small” effects. However, readers are able and encouraged to explore specific associations in more detail via the online tool and publicly-available full set of summary statistics (that can be downloaded from the online tool). This is explained in the main text: “To aid visual comparisons between binary and continuous outcome results, we converted the log odds ratios for binary outcomes to Cohen’s D (27) (standardised mean difference) which is a unitless measure of effect (equation provided in Supplementary File 3). Effect estimates are presented in their raw format (i.e. not as Cohen’s D) in the full set of results.” We have now added an additional justification for the threshold: “To succinctly compare models in this paper, we categorised results by applying commonly used thresholds: FDR-adjusted P<0.05 to define “statistical significance”, and D>0.2 to define anything other than a “small” effect.”

Finally, it would be reasonable to add some sensitivity analyses/I^2 statistics to the manuscript for exposure/outcome combinations that are highlighted in the manuscript.

- Response: The exposure/outcome combinations highlighted in the manuscript are:
  - The association between maternal smoking during pregnancy and child externalising traits, where we have already reported the I^2^ and heterogeneity P-value: “This shows that three cohorts (ALSPAC, MCS, MoBa) contributed to the meta-analysis, with a sample size of 40,014 (n smokers = 2971; 7%), and low heterogeneity (I^2^ = 23.4; heterogeneity P-value = 0.27).”
  - The exposure-outcome combinations for which there is most evidence for causal effects according to our triangulation analysis. For each exposure/outcome combination for each parent, there are between 14 and 30 models, so it is not straightforward to summarise these, and the heterogeneity statistics, in the main text. However, we have now added the summary statistics, including I^2^ and heterogeneity P-values, for all relevant models to Supplementary File 5 and have highlighted this in the main text “(full results and heterogeneity statistics presented in Supplementary File 5)”.
- In addition, all I^2^ and heterogeneity P-values are available in the online tool (under “deep dive” and are included in the full set of summary statistics that can be downloaded from the online tool.

**Comments from the reviewers:**

**Reviewer #1:**

The paper presents a comprehensive analysis using multiple datasets looking at parental influences on child health. The paper is generally well written and presented, and could provide a valuable resource for researchers in the field along with the online tool provided. The main strengths and limitations of the study are well summarised in the Discussion. My main comments are around the causal analysis.

The Mendelian randomization results are obtained by including a GRS in the model as an exposure. This amounts to a one-sample approach for Mendelian randomization. As indicated in the paper, many of the instruments are very weak, and in a one-sample setting, weak instrument bias can be in any direction, not necessarily toward the null. In Supplementary File 3 it is stated that bias due to weak instruments will be toward the null, however this is not necessarily the case here. It is further possible that the exclusion restriction assumption is violated due to genetic pleiotropy.

- Response: we have now adjusted the discussion of violation of MR assumptions and their likely impact on results (supplementary file 3) as follows: “Violation of the relevance assumption would introduce ‘weak instrument bias’, which could bias estimates in either direction. The exclusion assumption could also be violated by genetic pleiotropy (the GRS affecting multiple phenotypes), which may bias estimates in either direction depending on whether pleiotropic pathways act in the same or opposite direction as the exposure. In addition, given that parents transmit 50% of their genetic information to their children, some of the estimated effect may be driven by direct genetic effects in the child rather than a causal effect of the parental exposure. Therefore, without adjustment for child’s GRS (i.e. Model 2a/2b), the effect estimate may be overestimated away from the null (2) (although the direction depends on the relationship between the transmitted genetic effects and the outcome). However, adjusting for child’s GRS (Model 4a) may introduce collider bias, which can bias estimates in an unpredictable direction. Additional adjustment for the other parent’s GRS (i.e Model 4b) may help to mitigate bias arising from conditioning on child’s GRS. However, because genetic data are only available for partners in one cohort, these analyses are restricted to smaller samples and consequently have lower power. Therefore, when triangulating evidence, we use Model 2a, but heed caution that these estimates may be biased.

It would be recommended therefore to include some weak instrument and pleiotropy robust methods to mitigate these issues (see, for example, PMID 37325194 for examples of possible approaches). Such methods generally require genetic associations with the outcomes. Otherwise, it may be advisable to temper the language around this approach as testing for causal effects, rather they are controlling for confounding due to genetic factors.

- Response: We thank the reviewer for this helpful suggestion. Our analyses were based on polygenic risk score (GRS)–outcome associations (i.e. reduced-form models), and we did not perform formal instrumental variable estimation (e.g. Wald ratio or two-stage least squares). As such, these methods are not directly applicable in our setting, which does not involve SNP-level genetic associations with the outcomes. In line with the reviewer’s recommendation, we have revised the manuscript to clarify the role of the GRS analyses within a Mendelian randomisation framework and to avoid implying that we estimated causal effects using MR. Specifically, we now describe these analyses as genetically informed (reduced-form) approaches and emphasise that they contribute one line of evidence within a triangulation framework, rather than providing standalone causal effect estimates. We have also updated the limitations section to reflect the implications of weak genetic instruments in this context.
  - In supplementary file 3: “These analyses correspond to a “reduced-form” mendelian randomization, i.e., describing the association between the GRS and the outcome. We did not calculate an MR estimator (e.g. the Wald ratio) to describe the magnitude of any causal effect.”, and “We perform a “reduced-form” MR that assesses the association between the GRS and the outcome (but does not divide this association by the GRS-exposure association to calculate the Wald estimate). Generally, the power for genetic analyses is lower than for observational analyses because the sample size with genetic data is lower and/or the GRS explains only a limited proportion of variance in the exposure. Therefore, confidence intervals are likely to be wide. However, within our triangulation framework, we pre-specify that estimates that are directionally consistent with the equivalent MVR analysis and have confidence intervals that do not cross the null provide suggestive evidence consistent with a causal effect. As with all causal inference approaches we have applied, this is intended as a pragmatic definition, rather than definitive evidence of causality”
- In the abstract we now refer to “a Mendelian randomization-informed approach using genetic risk scores”
- In Table 2, we have updated the title and final column heading from referring to “Criteria for evidence of a causal effect” to “Pragmatic criteria for suggestive evidence of a causal effect” and we have changed the MR method to “Mendelian randomization (MR) informed approach using parental genetic risk scores (GRS)”
- In the methods: “We calculated genetic risk scores (GRS) for parental smoking, alcohol, and coffee consumption to incorporate genetically informed analyses within a Mendelian randomization framework [21]. Specifically, GRS were used as proxies for these behaviours in reduced-form (genome-outcome) analyses.”
- In the Results: “For most exposure-outcome combinations, the direction of association is consistent between the equivalent observational (MVR) and GRS (MR-informed) analyses”
- In the Discussion: “Finally, some GRSs were only weakly associated with the behaviour they were intended to represent (see Supplementary File S1 for F-statistics). This means that the GRSs explained only a small proportion of the variance in the behaviour, which is likely to reduce the precision of the GRS-outcome estimates and limit their informativeness within the triangulation framework. We derived our GRSs using genome-wide significant variants identified in previous genome-wide association studies (GWAS) to help ensure that they specifically reflected the behaviours of interest; however, the strength of these scores depends on the sample size and power of the original GWAS. For smoking, alcohol, and caffeine consumption, we used the largest GWASs available at the time to maximise power. Including a wider set of genetic variants may capture more of the variation in these behaviours, but could reduce specificity and increase the risk of capturing pleiotropic pathways. Future research could explore alternative GRS construction approaches and conduct additional sensitivity analyses to assess the robustness of genetically informed results [58].”

A further minor point is that the second Mendelian randomization assumption is incorrectly stated: it is that the genetic instrument is not associated with any confounders of the exposure-outcome relationship.

- Response: We have now corrected this where it is stated in Supplementary File S3: “the GRS is not associated with any confounders of the exposure-outcome relationship”

**Reviewer #2:**

I am grateful for the opportunity to review this paper. It is a very important and timely manuscript and I have no hesitation in saying it is exactly the kind/calibre of study I would expect to see in a journal such as PLoS Medicine.

The authors systematically examined parental smoking, alcohol, and caffeine use, alongside socioeconomic position, across four large cohorts and found that associations with child health were generally small, but more importantly found stronger evidence for socioeconomic influences than for parental behaviours. This is an ambitious and rigorous study, drawing on harmonised data and multiple causal inference approaches. The methods are presented with transparency and clear acknowledgement of limitations. The findings have important implications for both policy and future research, shifting focus beyond maternal pregnancy effects to partners and wider social determinants.

I also want to highlight the freely accessible EPoCH Explorer web app: I explored it myself and found it to be a genuinely useful (and quite fun!) tool for researchers, and I sincerely thank the authors for making it openly available.

Recommendations/points for consideration

1. Although it has long been suspected that socioeconomic conditions matter at least as much as parental behaviours in shaping child health, I am not aware of previous studies that have systematically tested this within a DOHaD framework; the authors should make clearer in the Introduction and Discussion that this is, to my knowledge, the first large-scale comparison of its kind (if it is not the first study pitting personal behaviours vs SES on this outcomes, it would be useful to know what other studies found)

- Response: We thank the reviewer for their kind remarks about our work. We have now added: to the Introduction: “This is, to our knowledge, the first largescale, systematic comparison of these effects“, and to the Discussion: “To our knowledge, few studies have directly compared the relative contributions of parental behaviours and socioeconomic conditions within a DOHaD framework, particularly at this scale.”

2. The heavy reliance on complete-case analysis raises the possibility of selection bias if the missingness of data was not random - I think it would be important to acknowledge this explicitly as a limitation.

- Response: We acknowledge missing data as a limitation in the Discussion, but we have now expanded this: “Some of the variation in sample size across analyses arises due to missing data within collected variables. Due to the number and complexity of our analyses, we chose to use a complete-case approach and did not employ multiple imputation techniques. The exclusion of participants with incomplete data may have introduced bias, particularly if the data are not missing at random; in such cases, multiple imputation would also be unlikely to fully address this bias. This limitation may affect the representativeness of the sample, reduce precision, and potentially induce collider bias if missingness is related to both exposures and outcomes.”

3. It would be useful to know the potential scale and impact of including non-biological partners in the analyses, particularly for the Mendelian randomisation work where biological paternity is central to the assumptions.

- Response: Our previous work (<https://pubmed.ncbi.nlm.nih.gov/39465608/>; <https://pubmed.ncbi.nlm.nih.gov/18336062/>) has shown that the impact is minimal. This is described in the Discussion: “The inclusion of non-biological partners could mean that mothers are more genetically related to study children than partners are. Although this might bias partner estimates (in either direction depending on exposure/outcome), sensitivity analyses have shown that the bias is minimal, even with implausibly high simulated rates of non-paternity (55). Similarly, higher rates of missing data for partners relative to mothers has the potential to introduce bias, particularly as we have previously shown that those partners who are recruited tend to be of a higher SEP than those who are not (56). However, our previous simulation study provides confidence that any resulting selection bias on the partner or partner-adjusted maternal estimate was likely to be small (56).”
- For the MR analyses, all partners were the biological fathers. We have clarified this in the main text methods: “We had access to genetic data for mothers in ALSPAC, BiB and MoBa, and partners (confirmed biological fathers in this instance) in MoBa”, and Discussion: “It should also be noted that for our GRS analyses (where biological paternity is central to the assumptions), all included partners *were* the biological fathers, as confirmed by the genetics data.”

4. Genetic risk scores and instrument strength are far from my area of expertise, but nor will they be clear to most readers; a brief plain-language explanation of what a "weak instrument" means in practice, and why it matters for interpretation, would improve accessibility of the methods and results.

- Response: We have now added some short explanations to the main text:
  - In the methods: “GRS were calculated using PLINK V1.9, using genetic variants (SNPs) identified in large scale genomewide association studies (GWAS) of number of aggregated alcoholic drinks consumed per week (22), number of cups of coffee consumed per day (23), and four smoking-related traits: age of initiation or regular smoking, ever vs never been a regular smoker, cigarettes per day, smoking cessation (current vs former smoker) (22). Scores were weighted by the effect estimates reported in the corresponding GWAS and converted to standard deviation units (z scores) to facilitate comparing effects across different scores. F-statistics for instrument strength – that is, the strength of the association between each GRS and the exposure it is intended to represent – are shown in Supplementary File 1.”
  - In the results: “For most exposure-outcome combinations, the direction of association is consistent between the equivalent MVR and MR analyses. However, for caffeine consumption, this is not always the case, which could reflect residual confounding in the observational (MVR) estimates, or bias in the MR results if the GRS is a “weak instrument” – that is, only weakly related to the reported exposure (see Supplementary File 1 for F-statistics for instrument strength).” In the Discussion, we reworded and expanded for clarity: “Finally, some GRSs were only weakly associated with the behaviour they were intended to represent (see Supplementary File 1 for F-statistics). This means that the GRSs explained only a small proportion of the variance in the behaviour, which can make the MR estimates less precise or potentially biased. We derived our GRSs using the top genetic variants from previous genomewide association studies (GWAS) to help ensure that they specifically reflected the behaviours of interest, but the strength of the GRS depends on the power of the original GWAS. For smoking, alcohol, and caffeine consumption, we used the largest GWASs available at the time to maximise power. It is possible that including a wider set of genetic variants in our GRSs could have captured more of the variation in these behaviours, though this would likely reduce specificity.”

5. Not all analyses were multi-cohort (I think), and I found it difficult to determine which findings were based on pooled evidence and which on a single study; while this may not be central to the overall message, it is probably worth a sentence in the limitations section so readers are aware in case they miss this.

- Response: We thank the reviewer for highlighting that this was not clear. We had explained this as follows:
  - In the first paragraph of the results, we state: “For our standard models 2a and 2b (unstratified by sex, adjusted for the standard set of covariates), the number of analyses involving more than one cohort was 8955/29146 (31%) for model 2a and 7760/28171 (28%) for model 2b.”
  - Supplementary File 5 contains a detailed summary of the number of cohorts and sample sizes for each model.
  - In the Discussion, in the first line of the Limitations, we state “Despite our attempt to harmonise variables across studies, differences in availability of data meant that up to two thirds of our analyses were conducted within a single cohort”.
- In addition, we have now added the results for each model included in the triangulation analysis for exposure-outcome combinations showing the strongest evidence for exposure to Supplementary File 5 (tables 2 and 3), and the final column includes the number of cohorts that contributed to the analysis.

6. Certain important modifiers and mediators, such as breastfeeding, housing conditions, and vaccine uptake, are not discussed but may be relevant to interpreting both exposures and outcomes in this field (they are certainly hot topics) ; even if not available in the cohorts, a short note in the Limitations section would help readers place the findings in the wider context.

- Response: We have now added to the Limitations: “In addition, there are many other potential confounders, modifiers, and mediators of the relationships we studied, but we chose to focus on those that we felt would be most important and/or had been measured well and consistently between cohorts. Future research could explore the role of factors such as breastfeeding, housing conditions, early life infection, and vaccine uptake.”

Overall, this is a substantial and well-conducted piece of work with high relevance for both research and policy. The findings may indeed be regarded as hugely influential because the headline (that socioeconomic conditions show stronger associations with child health than specific parental health behaviours) is important to many stakeholders and policymakers. Also the open resource provided will be of real value to the researchers in the field.


Ian Sinha, Alder Hey Children's Hospital, Liverpool, UK

**Reviewer #3:**

Thank you for the opportunity to review this article. The EPOCH platform seems to provide a unique and ambitious tool that could expand the exploration of associations related to factors influencing the early stages of life course health.

The open access design offers transparency and the opportunity for the addition of further evidence from cohorts in contexts that vary from the four provided to date.
The explanation of the need for the  tool and its strengths regarding additional analyses are well described.

The concept that SES is a complex construct correlated with health outcomes is not novel. DOHaD evidence explores the complexity of the interplay between genetic, environmental, socio-political, and lifestyle factors within and across generations.
Is the contested issue that the authors are pointing to the oversimplification of association evidence in a complex system? If so, maybe it would be sensible to make this clearer? Even smoking, which the EPoCH evidence clearly points to as causal, cannot be dissociated from SES.

- Response: We thank the reviewer for highlighting that this wasn’t clear. We have now added:
  - To the conclusion of the abstract: “Our findings suggest that wider familial socioeconomic conditions may be a more important determinant of child health than specific parental health behaviours prenatally. Interventions to improve population health may be most effective if they target wider social inequalities, rather than individual behaviours and mothers specifically.”
  - To the introduction: “The Developmental Origins of Health and Disease (DOHaD) hypothesis posits that environmental exposures in early life, including those that occur prenatally, can affect long-term health (1). Traditionally, the field has focused on aspects of the maternal environment in and around pregnancy, including how a mother’s health behaviours might affect her fetus *in utero* (i.e. “maternal pregnancy effects”) (2). Despite the fact that these behaviours are socially-patterned and likely to be correlated with paternal and postnatal exposures, there has been much less of a focus on these other types of exposure (2–4). This makes it difficult to test (rather than merely reinforce) the DOHaD hypothesis, and to challenge implicit and deeply-held assumptions about the causal primacy of maternal pregnancy effects (2). It also restricts our ability to disentangle specific causal effects from the wider system in which they occur and to understand whether efforts to improve offspring health would be most effective if they were directed towards wider social determinants rather than specific behaviours of individual parents.”
  - In the discussion: “Our findings suggest that there is no strong scientific rationale to focus exclusively on maternal influences. Future DOHaD research should therefore avoid oversimplifying evidence on maternal pregnancy effects within a complex, socially patterned system, and instead broaden their scope to study data on partners, postnatal exposures, and wider social determinants of health.”

There is no question that the tool has enormous potential and could be very useful in supporting deeper understanding of the complexity of factors associated with long-term health outcomes. The current study offers data up to age 8 - well before many of the later-life outcomes are emerging.

- Response: We chose to focus on childhood outcomes (up to age 11) in this study, although we acknowledge that some outcomes may emerge later. We have now highlighted in the abstract that the outcomes are measured “up to age 11” and added to the Limitations: “EPoCH focused on outcomes in childhood (up to age 11), and we did not examine outcomes later in life, although we acknowledge that some outcomes may emerge later. This could be a focus of future largescale systematic explorations.”

Some comments within the limitations on factors beyond the three important ones chosen could also be interacting within the system to influence outcomes. Maternal stress (which is of course associated with SEs) seems to be a clear omission - albeit challenging to measure.

- Response: We agree that there are other exposures that could and should be studied, but in this project we chose to focus on potentially modifiable health behaviours (as mentioned in the Methods, “To maximise the relevance to public health policy, we focused on parental health behaviours that are potentially modifiable during pregnancy”). We have now added to the Limitations: “In addition, there are many other potential confounders, modifiers, and mediators of the relationships we studied, but we chose to focus on those that we felt would be most important and/or had been measured well and consistently between cohorts. Future research could explore the role of factors such as breastfeeding, housing conditions, early life infection, and parental psychological stress.”

It would also be helpful to see some comment on the limitations of the age to which the data reaches (age 8) re the associations of 'later life' health outcomes.

- Response: We do not use the term “later life outcomes” in our manuscript – we use the more appropriate “child health outcomes” – so as to avoid over-extrapolating our results. As previously noted, we have now added to the Limitations: “EPoCH focused on outcomes in childhood (up to age 11), and we did not examine outcomes later in life, although we acknowledge that some outcomes may emerge later. This could be a focus of future largescale systematic explorations.”

**Reviewer #4:**

Many thanks for the opportunity to review this paper.
This is an ambitious, well-executed high-throughput DOHaD paper that will be very valuable to the field for two reasons: (i) it systematically benchmarks a large set of prenatal parental behaviours (maternal and partner) against multiple outcomes using harmonised multicohort analyses (with meta-analysis where possible), and (ii) it operationalises transparent evidence triangulation via an open, interactive web tool (EPoCH Explorer) with downloadable summary statistics and code.

The main scientific contribution is not a single novel causal claim, but a disciplined recalibration of expectations: most parental prenatal behaviour-child outcome associations are small and not robust after multiple testing, whereas socioeconomic position (SEP) shows consistently stronger associations across domains.

Minor points for consideration:
1.Clarify the inferential target (avoid "evidence of no effect" interpretation). The central message, which is limited widespread evidence for large prenatal behaviour effects, could be misread as disproving prenatal behavioural influences outcomes. Strengthen the framing in Abstract/Discussion to distinguish: (i) genuinely small effects after confounding control/multiple testing from (ii) null results driven by measurement error, limited partner data, or imprecision in specific exposure-outcome strata.

- Response: We thank the reviewer for this thoughtful point. Our intention was not to imply evidence of no effect, but rather to summarise the lack of strong evidence for widespread or large effects within the scope of our analyses. We have been careful throughout to frame results in terms of the strength of evidence rather than the absence of effects. For example, in our results section, we state “Note that a lack of evidence does not indicate evidence against a causal effect during pregnancy; it may reflect limited data availability or differences in assumptions across contexts.” We have now reiterated this point in the Discussion: “In this largescale systematic exploration – of four types of exposure in both parents at up to nine timepoints, on 72 child outcomes at up to four ages – we did not find strong evidence for widespread or large magnitude effects of parental health behaviours on child health and wellbeing (although we note that a lack of evidence does not indicate evidence against a causal effect; it may reflect issues such as low power, measurement error, and missing data – see Limitations)”
- We agree that distinguishing between genuinely small effects and null results arising from measurement error or imprecision is an important consideration. However, we do not believe this distinction can be clearly made. We have therefore summarised all results using a measure of effect magnitude (standardised effect estimate (Cohen’s D) using a widely-used threshold of <0.2 to distinguish small from greater-than-small effects), as well as a measure of precision (FDR-adjusted P-value <0.05). The raw effect estimates and 95% confidence intervals are also available. We present these for up to 8 different models, with different adjustment sets, to explore the impact of confounder adjustment on effect sizes and precision, which we summarise in Supplementary File 5. We also describe the reduction in sample size (and therefore power) introduced by progressive adding of covariates to the models. The full set of summary statistics is provided via EPoCH Explorer.

2. The authors note complete-case analysis and potential MNAR bias, but the scale of analyses amplifies concerns about selection (partner exposure availability, outcome follow-up). Could more detail or comment around missingness/attrition summary by cohort and domain be provided.

- We now expanded the relevant section of the Discussion to read: “Some of the variation in sample size across analyses arises due to missing data within collected variables. Due to the number and complexity of our analyses, we chose to use a complete-case approach and did not employ multiple imputation techniques. The exclusion of participants with incomplete data may have introduced bias, particularly if the data are not missing at random; in such cases, multiple imputation would also be unlikely to fully address this bias. This limitation may affect the representativeness of the sample, reduce precision, and potentially induce collider bias if missingness is related to both exposures and outcomes.”
- In addition, Supplementary file 6 contains columns for the number and percentage missing for every variable (exposure, covariate, outcome) in each cohort. We have now highlighted this in the main text: “Supplementary File 6 contains a detailed breakdown of sample characteristics by cohort, including the distribution and level of missingness for each variable.”

3. Partner exposure is a strong negative control for maternal intrauterine effects only when key assumptions hold. For outcomes where partner pathways are plausible (passive smoke exposure; shared household diet; parenting stress; postnatal continuation of behaviours), similarity does not necessarily imply "non-causal." Could the authors add an outcome typology and explicitly flag domains where partner negative controls are less clear?

- Response: We thank the reviewer for highlighting a common misconception of parental negative control designs. We have now clarified the hypothesis that the parental negative control comparison (and other causal inference approaches included in our triangulation analysis) is intended to address in the Methods: “Our analyses allowed us to apply multiple causal inference approaches to strengthen causal interpretation of associations. In the analyses presented in this paper, the causal hypothesis was that there is a direct effect of the parental exposure *during pregnancy*.” In addition, this is already described in Supplementary File 3 (“EPoCH analyses were designed to enable us to garner evidence from multiple causal inference approaches to test the causal hypothesis for health behaviours *during pregnancy* affecting child health via an *in-utero* mechanism”).
- The existence of potential shared confounders (like passive smoking via the partner, or shared household diet) or common post-pregnancy exposures (like parenting stress or postnatal continuation of behaviours) does not invalidate the use of the paternal exposure as a negative control to address this hypothesis – in fact, the influence of these shared covariates are precisely what the analysis is aiming to overcome. For example, in the case of partner smoking, if there were an intrauterine effect of tobacco exposure, we would expect associations between maternal smoking and offspring outcomes to be stronger than associations between partner smoking and offspring outcomes because the maternal effect would be operating via a direct intrauterine mechanism while the partner effect would be operating only indirectly via passive smoke exposure. This is explained in Supplementary File 3, and we do not feel there is space to describe this in more detail in the manuscript without going into a similar level of detail about the other approaches, but we will of course take the Editor’s advice on this.
- Within parental comparisons, the outcome is always the same, so the validity of this approach does not depend on the outcome and therefore an outcome typology would not be useful.

4. FDR at 5% plus Cohen's D>0.2 is pragmatic, but the D threshold may be questioned across heterogeneous outcomes. Could the authors comment on sensitivity summaries using alternative thresholds (e.g., D>0.1) so aid justifying the approach?

- Response: We explore results using Cohen’s D because it provides a standardised effect estimate that enables us to compare effect sizes across different scales and types of outcome (core to our aim to provide a systematic exploration of our hypothesis), and the threshold selected is commonly used to indicate “small” effects. However, readers are able and encouraged to explore specific associations in more detail via the online tool and publicly-available full set of summary statistics (that can be downloaded from the online tool). This is explained in the main text: “To aid visual comparisons between binary and continuous outcome results, we converted the log odds ratios for binary outcomes to Cohen’s D (27) (standardised mean difference) which is a unitless measure of effect (equation provided in Supplementary File 3). Effect estimates are presented in their raw format (i.e. not as Cohen’s D) in the full set of results.” We have now added an additional justification of the threshold: “To succinctly compare models in this paper, we categorised results by applying commonly used thresholds: FDR-adjusted P<0.05 to define “statistical significance”, and D>0.2 to define anything other than a “small” effect.”

5. The fixed-effects choice due to few cohorts is understandable, but readers may want clarity about heterogeneity (measurement, cohort composition). Could authors provide heterogeneity indicators and/or a small sensitivity comparison (fixed vs random) for key exposure-outcome pairs, or explicitly flag where heterogeneity is non-trivial and conclusions should be cautious.

- Response: The exposure/outcome combinations highlighted in the manuscript are:
  - The association between maternal smoking during pregnancy and child externalising traits, where we have already reported the I^2 and heterogeneity P-value: “This shows that three cohorts (ALSPAC, MCS, MoBa) contributed to the meta-analysis, with a sample size of 40,014 (n smokers = 2971; 7%), and low heterogeneity (I^2^ = 23.4; heterogeneity P-value = 0.27).”
  - The combinations where there is most evidence for causal effects according to our triangulation analysis. For each exposure/outcome combination for each parent, there are between 14 and 30 models, so it is not straightforward to summarise these, and the heterogeneity statistics, in the main text. However, we have now added the summary statistics, including I^2^ and heterogeneity P-values, for all relevant models to Supplementary File 5 and have highlighted this in the main text “(full results and heterogeneity statistics presented in Supplementary File 5)”.
- In addition, all I^2^ and heterogeneity P-values are available in the online tool (under “deep dive” and full set of summary statistics that can be downloaded from the online tool.

6. The cohorts are predominantly European/Northern high-income settings; add a clearer statement on applicability to LMIC contexts and settings with different exposure distributions, SEP structures, and competing risks.

- Response: “We have slightly clarified our statement on this in the Limitations: The generalisability of our findings is also constrained by the low ethnic and socioeconomic diversity across the cohorts, except for BiB. In addition, all cohorts are based in high-income countries in Northern Europe, so it is unclear whether our findings apply to other populations and settings.”

**General editorial requests:**

*       Please upload any figures associated with your paper as individual TIF or EPS files with 300dpi resolution at resubmission; please read our figure guidelines for more information on our requirements:

- We have used NAAS to format the figures correctly

*       We expect all researchers with submissions to PLOS in which author-generated code underpins the findings in the manuscript to make all author-generated code available without restrictions upon publication of the work. In cases where code is central to the manuscript, we may require the code to be made available as a condition of publication. Authors are responsible for ensuring that the code is reusable and well documented. Please make any custom code available, either as part of your data deposition or as a supplementary file.

- We have added links to GitHub and Zenodo

*    Data availability statement: Please include a statement on the criteria that researchers need to fulfill in order to be granted access to the data.
- Complete

*   Ethics statement: I see that for each cohort there was consent from an ethics committee. However, please provide an ethics statement in the method on whether this particular study is approved by an ethics committee. You can include in this statement that the descriptions/ethics approval for each cohort can be found in the supplements. Please also ensure that for each cohort you describe whether participants provided oral or written consent.

- Complete

FORMATTING - GENERAL
*       Abstract: Please structure your abstract using the PLOS Medicine headings (Background, Methods and Findings, Conclusions). Please combine the Methods and Findings sections into one section.

- Complete

*       At this stage, we ask that you include a short, non-technical Author Summary of your research to make findings accessible to a wide audience that includes both scientists and non-scientists.

- Complete

*       Please express the main results with 95% CIs as well as p values. When reporting p values please report as p<0.001 and where higher as the exact p value p=0.002, for example. Throughout, suggest reporting statistical information as follows to improve clarity for the reader "22% (95% CI [13%,28%]; p</=)". Please be sure to define all numerical values at first use.

- Complete

*       Please include page numbers and line numbers in the manuscript file. Use continuous line numbers (do not restart the numbering on each page).

- Complete

*       Please cite the reference numbers in square brackets. Citations should precede punctuation.

- Complete

FIGURES AND TABLES
*       Please provide titles and legends for all figures and tables (including those in Supporting Information files).

- Complete

*       Please define all abbreviations used in each figure/table (including those in Supporting Information files).

- Complete

SUPPLEMENTARY MATERIAL
*       Please note that supplementary material will be posted as supplied by the authors. Therefore, please amend it according to the relevant comments outlined here.

- Complete

*       Please cite your Supporting Information as outlined here: <https://eur03.safelinks.protection.outlook.com/?url=https%3A%2F%2Fjournals.plos.org%2Fplosmedicine%2Fs%2Fsupporting-information&data=05%7C02%7Cg.c.sharp%40exeter.ac.uk%7C5ce76ec2af41455a3cd408de6f10ebce%7C912a5d77fb984eeeaf321334d8f04a53%7C0%7C0%7C639070315643426452%7CUnknown%7CTWFpbGZsb3d8eyJFbXB0eU1hcGkiOnRydWUsIlYiOiIwLjAuMDAwMCIsIlAiOiJXaW4zMiIsIkFOIjoiTWFpbCIsIldUIjoyfQ%3D%3D%7C0%7C%7C%7C&sdata=ZV8Trn%2BffKCZySFrfFtFtdM6xyXPXtIpKfqstneG0Gc%3D&reserved=0>

- Complete

REFERENCES
*       PLOS uses the numbered citation (citation-sequence) method and first six authors, et al.

- Complete

*       Please ensure that journal name abbreviations match those found in the National Center for Biotechnology Information (NCBI) databases (<https://eur03.safelinks.protection.outlook.com/?url=http%3A%2F%2Fwww.ncbi.nlm.nih.gov%2Fnlmcatalog%2Fjournals&data=05%7C02%7Cg.c.sharp%40exeter.ac.uk%7C5ce76ec2af41455a3cd408de6f10ebce%7C912a5d77fb984eeeaf321334d8f04a53%7C0%7C0%7C639070315643450535%7CUnknown%7CTWFpbGZsb3d8eyJFbXB0eU1hcGkiOnRydWUsIlYiOiIwLjAuMDAwMCIsIlAiOiJXaW4zMiIsIkFOIjoiTWFpbCIsIldUIjoyfQ%3D%3D%7C0%7C%7C%7C&sdata=B7zQ2ELzdX30aWa9%2FMeNoZtHDd82FmGAP5zprjEK9kc%3D&reserved=0>), and are appropriately formatted and capitalised.

- Complete

*       Where website addresses are cited, please include the complete URL and specify the date of access (e.g. [accessed: 12/06/2023]).

- Complete

*       Please also see <https://eur03.safelinks.protection.outlook.com/?url=https%3A%2F%2Fjournals.plos.org%2Fplosmedicine%2Fs%2Fsubmission-guidelines%23loc-references&data=05%7C02%7Cg.c.sharp%40exeter.ac.uk%7C5ce76ec2af41455a3cd408de6f10ebce%7C912a5d77fb984eeeaf321334d8f04a53%7C0%7C0%7C639070315643726945%7CUnknown%7CTWFpbGZsb3d8eyJFbXB0eU1hcGkiOnRydWUsIlYiOiIwLjAuMDAwMCIsIlAiOiJXaW4zMiIsIkFOIjoiTWFpbCIsIldUIjoyfQ%3D%3D%7C0%7C%7C%7C&sdata=Fd7cWzH2U8Wtt3%2FRnDtU2ieP1fSBx0K4ui4bxFn3c8Y%3D&reserved=0> for further details on reference formatting.

- Complete

OBSERVATIONAL STUDIES

*       Abstract: Please include the study design, population and setting, number of participants, years during which the study took place (enrollment and follow up), length of follow up, and main outcome measures.

- Complete

*       Please state in the Methods section whether the study had a prospective protocol or analysis plan. If a prospective analysis plan (from your funding proposal, IRB or other ethics committee submission, study protocol, or other planning document written before analyzing the data) was used in designing the study, please include the relevant document(s) with your revised manuscript as a Supporting Information file to be published alongside your study and cite it in the Methods section. A legend for this file should be included at the end of your manuscript. If no such document exists, please make sure that the Methods section transparently describes when analyses were planned, and when/why any data-driven changes to analyses took place. Changes in the analysis, including those made in response to peer review comments, should be identified as such in the Methods section of the paper, with rationale.
- Complete


MENDELIAN RANDOMIZATION STUDIES

*       Please ensure that the study is reported according to the STROBE-MR guideline (<https://eur03.safelinks.protection.outlook.com/?url=https%3A%2F%2Fwww.equator-network.org%2Freporting-guidelines%2Fstrobe%2F&data=05%7C02%7Cg.c.sharp%40exeter.ac.uk%7C5ce76ec2af41455a3cd408de6f10ebce%7C912a5d77fb984eeeaf321334d8f04a53%7C0%7C0%7C639070315643805488%7CUnknown%7CTWFpbGZsb3d8eyJFbXB0eU1hcGkiOnRydWUsIlYiOiIwLjAuMDAwMCIsIlAiOiJXaW4zMiIsIkFOIjoiTWFpbCIsIldUIjoyfQ%3D%3D%7C0%7C%7C%7C&sdata=6uRMGypaUJs%2FOQAmyoVKrNdesZbZdVW0CPNWAxHdXRc%3D&reserved=0>) and include the completed STROBE-MR checklist as Supporting Information. Please add the following statement, or similar, to the Methods: "This study is reported as per the Strengthening the Reporting of Observational Studies in Epidemiology (STROBE) guideline, specific for mendelian randomization (S1 Checklist)." When completing the checklist, please use section and paragraph numbers, rather than page numbers.

- Complete

*       In the Introduction, please describe the exposure and the evidence for a potential causal relationship between exposure and outcome.

- This is not straightforward as we have three different types of exposure and 72 outcomes, but I think this may be redundant in our case because the whole study explores the evidence for potential causal relationships between the exposures and outcomes.

*       In the Methods, please explicitly state the 3 core instrumental variable assumptions for the main analysis (relevance, independence, and exclusion restriction), as well assumptions for any additional or sensitivity analysis.

- Complete (Described in Supplementary File 3)

*       In the Methods, please describe the MR estimator (e.g., 2-stage least squares, Wald ratio) and related statistics. Detail the included covariates and, in case of 2-sample MR, whether the same covariate set was used for adjustment in the 2 samples.

- Complete (Described in Supplementary File 3. We apply the principles of MR by calculating the “reduced-form” association (genetic variant and outcome) but we do not calculate an MR estimator.)

*       If you are presenting an instrumental variable estimate, please compare this to the conventional observational estimate.

- Complete (We do not present an IV estimate, but it is possible to compare the GRS-outcome to exposure-outcome estimates through EPoCH Explorer.)

*       Report the associations between genetic variant and exposure and between genetic variant and outcome, preferably on an interpretable scale.
- Complete (F-statistics for GRS-exposure; regression coefficients for GRS-outcome)

*       Report MR estimates of the relationship between exposure and outcome and the measures of uncertainty from the MR analysis, on an interpretable scale, such as odds ratio or relative risk per SD difference.

- Complete

*       If relevant, please consider translating estimates of relative risk into absolute risk for a meaningful time period.

- Not relevant – we do not estimate the magnitude of the causal effect using MR

*       Please consider including plots to visualize results (e.g., forest plot, scatterplot of associations between genetic variants and outcome vs between genetic variants and exposure).
- Complete (via EPoCH Explorer)

1) We ask that a manuscript source file is provided at Revision. Please upload your manuscript file as a .doc, .docx, .rtf or .tex.

- Complete

2) Please provide separate figure files in .tif or .eps format.

- Complete

3) We notice that your supplementary figures and tables are included in the manuscript file. Please remove them and upload them with the file type 'Supporting Information'. Please ensure that each Supporting Information file has a legend listed in the manuscript after the references list.

- Complete

4) Please refer to the following article for PLOS Biology's best-practice recommendations for data presentation: <https://eur03.safelinks.protection.outlook.com/?url=https%3A%2F%2Fjournals.plos.org%2Fplosbiology%2Farticle%3Fid%3D10.1371%2Fjournal.pbio.1002128&data=05%7C02%7Cg.c.sharp%40exeter.ac.uk%7Cdc5532cb64bd45c7e3f208de6fe22b4b%7C912a5d77fb984eeeaf321334d8f04a53%7C0%7C0%7C639071214334303950%7CUnknown%7CTWFpbGZsb3d8eyJFbXB0eU1hcGkiOnRydWUsIlYiOiIwLjAuMDAwMCIsIlAiOiJXaW4zMiIsIkFOIjoiTWFpbCIsIldUIjoyfQ%3D%3D%7C0%7C%7C%7C&sdata=PmzkGl27bQvJubqP%2FLHpaz2iAyLRwUUd5ELPVkkgc2I%3D&reserved=0>.

- Complete

Guidelines for statistical reporting, if relevant, may be found here: <https://eur03.safelinks.protection.outlook.com/?url=https%3A%2F%2Fjournals.plos.org%2Fplosbiology%2Fs%2Fbest-practices-in-research-reporting%23loc-statistical-reporting&data=05%7C02%7Cg.c.sharp%40exeter.ac.uk%7Cdc5532cb64bd45c7e3f208de6fe22b4b%7C912a5d77fb984eeeaf321334d8f04a53%7C0%7C0%7C639071214334323050%7CUnknown%7CTWFpbGZsb3d8eyJFbXB0eU1hcGkiOnRydWUsIlYiOiIwLjAuMDAwMCIsIlAiOiJXaW4zMiIsIkFOIjoiTWFpbCIsIldUIjoyfQ%3D%3D%7C0%7C%7C%7C&sdata=GbBtxB%2B%2FD%2BQu%2Bv%2Fgk5FsvNUQuO4ljaEujOsdBz7mynI%3D&reserved=0>.

- Complete

5) Figure 4A, SUPPLEMENTARY FILE 7 (Figures 1-6 and 9) contains screenshots. We are not permitted to publish these under our CC BY 4.0 license; websites are usually intellectual property and are copyrighted. This includes peripheral graphics of the web browser such as the [X] buttons. We ask that you please remove or replace it.

- The screen shots are from EPoCH Explorer, the website that we have created as part of this study. The paper describes the website, so screenshots help to illustrate its functionality. We don’t include any graphics from the browser. Please let us know how you would like to proceed with this.
