# Supplementary material for: Exploring parental prenatal influences on child health: A multicohort study and data visualisation tool
Source: PLoS Med. 2026 Jul 23;23(7):e1005153. doi: 10.1371/journal.pmed.1005153 (PMC13395330; doi:10.1371/journal.pmed.1005153)
Supplement: Attachment — Submitted filename: R2 Response.docx [file pmed.1005153.s013.docx]

* Please confirm that your title complies with PLOS Medicine's style. Your title must be nondeclarative and not a question. It should begin with main concept if possible. "Effect of" should be used only if causality can be inferred, i.e., for an RCT. Please place the study design ("A randomized controlled trial," "A retrospective study," "A modelling study," etc.) in the subtitle (ie, after a colon).

YES

* Please confirm that your abstract complies with our requirements, including format (three sections: Background, Methods and Findings, and Conclusions) and providing all the information relevant to this study type

YES

* Please ensure that all abbreviations are defined at first use throughout the text.

YES

* Please confirm that all numbers presented in the abstract are present and identical to numbers presented in the main manuscript text.

YES

* Please disclose any use of AI tools and technologies to the study or article content in the Methods, including the name(s) of any tool(s) used and a description of their use.

NO AI TOOLS WERE USED

* Please review your text for claims of novelty or primacy (e.g. 'for the first time') and remove this language. In addition, please check that any use of statistical terms (such as trend or significant) are supported by the data, and if not please remove them.

REMOVED ONE LINE THAT HAD BEEN SUGGESTED BY A REVIEWER

* Please remove the 'conclusions' subheading from the discussion. Please also remove any other subheadings from the discussion.

DONE

* Statistical reporting: Please revise throughout the manuscript, including tables and figures.

- Please report statistical information as follows to improve clarity for the reader ""22% (95% CI [13,28]; p</=)"".

- Please separate upper and lower bounds with commas instead of hyphens as the latter can be confused with reporting of negative values.

- Please repeat statistical definitions (HR, CI etc.) for each set of parentheses.

DONE

* Line 184-185: please check if this sentence is correct, and modify if necessary: “caffeine from tea, coffee, cola, and any of those sources” .

EDITED TO "caffeine consumed from tea, coffee and cola (both individually and combined)."

ABSTRACT:

* In the last sentence of the Abstract Methods and Findings section, please describe the main limitation(s) of the study's methodology.

ADDED: "Sample sizes and statistical power varied across outcomes and there was limited ethnic diversity in our study samples."

DATA AVAILABILITY:

*Thank you for providing the used code, as well as a Zenodo link. We need a permanent record of all the data. As the website could at some point be taken down for any reason, can you:

1) Confirm that all results can be generated with the provided code?

2) Can you provide the summary statistics in a different way, instead of only on the EPoCH Explorer? This could for instance be as a supplemental file, or be included in the code/Zenodo links?

YES. LINK TO SUMMARY STATISTICS NOW PROVIDED: https://doi.org/10.5281/zenodo.20490585

*Could you include your statement on Code availability in your Data availability statement (in the MS and the metadata)?

UPDATED IN THE MS AND SUPPLEMENTARY MATERIAL AND VIA THE PORTAL

ETHICS STATEMENT:

*Thank you for including your ethics statement. do you have approval numbers from the ALSPAC, BIB and MoBa executive board? If yes, please include these in your ethics statement.

ONLY FOR ALSPAC (B3123) - THIS IS NOW ADDED

FIGURES

* When a p value is given, please specify the statistical test used to determine it in the legend.

HAVE ADDED REGRESSION TO LEGENDS

OBSERVATIONAL STUDIES

* Thank you for including your analysis plan as a supplement. Am I correct that this was a prospective plan? Please state this (either way) early in the Methods section.

THIS IS ALREADY STATED UNDER OPEN SCIENCE AND REPRODUCIBILITY: "The general analysis plan was outlined prospectively in the funding proposal, which is included, along with a list of changes and their rationales, in Supplementary File S4."

a) If a prospective analysis plan (from your funding proposal, IRB or other ethics committee submission, study protocol, or other planning document written before analyzing the data) was used in designing the study, please include the relevant prospectively written document with your revised manuscript as a Supporting Information file to be published alongside your study, and cite it in the Methods section. A legend for this file should be included at the end of your manuscript.

ALREADY INCLUDED

b) If no such document exists, please make sure that the Methods section transparently describes when analyses were planned, and when/why any data-driven changes to analyses took place.

N/A

c) In either case, changes in the analysis-- including those made in response to peer review comments-- should be identified as such in the Methods section of the paper, with rationale.

ALREADY INCLUDED IN FILE S4.

* Thank you for including the STROBE checklist. Could you provide this as a separate file, rather than as part of the supplementary material?

I'M NOT SURE HOW TO ADDRESS THIS. DO YOU MEAN YOU DON'T WANT IT AS PART OF THE SUPPLEMENTARY MATERIAL? HOW DO I PROVIDE IT AS A SEPARATE FILE?

provide a short, non-technical Author Summary of your research to make findings accessible to a wide audience that includes both scientists and non-scientists. The Author Summary should immediately follow the Abstract in your revised manuscript. This text is subject to editorial change and should be distinct from the scientific abstract.

ALREADY INCLUDED

1) Thank you for stating "The funders had no role in any aspect of the design or execution of the study."

Please amend your detailed Financial Disclosure statement. This is published with the article, therefore should be completed in full sentences and contain the exact wording you wish to be published.

i). State what role the funders took in the study. If the funders had no role in your study, please state: “The funders had no role in study design, data collection and analysis, decision to publish, or preparation of the manuscript.”

EDITED IN MANUSCRIPT AND PORTAL

ii). If any authors received a salary from any of your funders, please state which authors and which funders.

UPDATED IN PORTAL
