# Supplementary material for: Exploring parental prenatal influences on child health: A multicohort study and data visualisation tool
Source: PLoS Med. 2026 Jul 23;23(7):e1005153. doi: 10.1371/journal.pmed.1005153 (PMC13395330; doi:10.1371/journal.pmed.1005153)
Supplement: Peer review history

# Editor report: Decision Letter 0

**DOI:** 10.1371/journal.pmed.1005153.r001

**Authors:** Richard Turner

27 Aug 2025

Dear Dr Sharp,

Thank you for submitting your manuscript entitled "Exploring parental prenatal influences on child health: a multicohort study and data visualisation tool" for consideration by PLOS Medicine.

Your manuscript has now been evaluated by the PLOS Medicine editorial staff, and I am writing to let you know that we would like to send your submission for external assessment.

However, we first need you to complete your submission by providing the metadata that are required for full assessment. To this end, please login to Editorial Manager where you will find the paper in the 'Submissions Needing Revisions' folder on your homepage. Please click 'Revise Submission' from the Action Links and complete all additional questions in the submission questionnaire.

For clinical studies, please upload a copy of your trial study protocol as a supporting information file. The study protocol should be the version submitted for approval to the institutional review board or ethics committee, should include any amendments to the study protocol, as well as the date of their approval by the institutional review or ethics committee. Please also detail any deviations from the study protocol in the Methods section of your manuscript. The editors will consider the protocol and study conduct prior to a final decision for external review.

Please re-submit your manuscript within two working days, i.e. by Aug 29 2025 11:59PM.

Login to Editorial Manager here: https://www.editorialmanager.com/pmedicine

Once your submission is complete, your paper will undergo a series of checks in preparation for full assessment.

Feel free to email us at plosmedicine@plos.org if you have any queries relating to your submission.

Kind regards,

Richard Turner, PhD

Consulting Editor, PLOS Medicine

plosmedicine@plos.org

# Peer review: Decision Letter 1

**DOI:** 10.1371/journal.pmed.1005153.r002

**Authors:** Richard Turner, Suzanne De Bruijn

18 Feb 2026

Dear Dr Sharp,

Many thanks for submitting your manuscript "Exploring parental prenatal influences on child health: a multicohort study and data visualisation tool" (PMEDICINE-D-25-02845R1) to PLOS Medicine. The paper has been reviewed by subject experts and a statistician; their comments are included below and can also be accessed here: [LINK]

As you will see, the reviewers thought this is an interesting and ambitious study, and applaud you for providing the online tool. However, they also had some concerns and suggestions. After discussing the paper with the editorial team and an academic editor with relevant expertise, I'm pleased to invite you to revise the paper in response to the reviewers' comments. We plan to send the revised paper to some or all of the original reviewers, and we cannot provide any guarantees at this stage regarding publication.

In addition to these revisions, you may need to complete some formatting changes, which you will receive in a follow up email. A member of our team will be in touch with a set of requests shortly. If you do not receive a separate email within a few days, please assume that checks have been completed, and no additional changes are required.

When you upload your revision, please include a point-by-point response that addresses all of the reviewer and editorial points, indicating the changes made in the manuscript and either an excerpt of the revised text or the location (eg: page and line number) where each change can be found. Please also be sure to check the general editorial comments at the end of this letter and include these in your point-by-point response. When you resubmit your paper, please include a clean version of the paper as the main article file and a version with changes tracked as a marked-up manuscript. It may also be helpful to check the guidelines for revised papers at http://journals.plos.org/plosmedicine/s/revising-your-manuscript for any that apply to your paper.

We ask that you submit your revision by Apr 22 2026 11:59PM. However, if this deadline is not feasible, please contact me by email, and we can discuss a suitable alternative.

Don't hesitate to contact me directly with any questions (sbruijn@plos.org).

Best regards,

Suzanne

Suzanne De Bruijn, PhD

Associate Editor

PLOS Medicine

sbruijn@plos.org

-----------------------------------------------------------

Comments from the editor:

We discussed some of the reviewer comments with our statistical reviewer. They mentioned that the missing data is a limitation, that should be highlighted (although they mention that it is briefly noted in the discussion). They also stated that the suggestion of Reviewer #4 to provide an indication of the level of missingness in the different cohort is reasonable.

Although we would encourage you to address all reviewer comments, I do add that our statistical reviewer did think that the D threshold is reasonable to use, and that additional analyses may not be necessary, as the online tool allows users to explore specific effects. Finally, it would be reasonable to add some sensitivity analyses/I^2 statistics to the manuscript for exposure/outcome combinations that are highlighted in the manuscript.

-----------------------------------------------------------

Comments from the reviewers:

Reviewer #1: The paper presents a comprehensive analysis using multiple datasets looking at parental influences on child health. The paper is generally well written and presented, and could provide a valuable resource for researchers in the field along with the online tool provided. The main strengths and limitations of the study are well summarised in the Discussion. My main comments are around the causal analysis.

The Mendelian randomization results are obtained by including a GRS in the model as an exposure. This amounts to a one-sample approach for Mendelian randomization. As indicated in the paper, many of the instruments are very weak, and in a one-sample setting, weak instrument bias can be in any direction, not necessarily toward the null. In Supplementary File 3 it is stated that bias due to weak instruments will be toward the null, however this is not necessarily the case here. It is further possible that the exclusion restriction assumption is violated due to genetic pleiotropy.

It would be recommended therefore to include some weak instrument and pleiotropy robust methods to mitigate these issues (see, for example, PMID 37325194 for examples of possible approaches). Such methods generally require genetic associations with the outcomes. Otherwise, it may be advisable to temper the language around this approach as testing for causal effects, rather they are controlling for confounding due to genetic factors.

A further minor point is that the second Mendelian randomization assumption is incorrectly stated: it is that the genetic instrument is not associated with any confounders of the exposure-outcome relationship.

Reviewer #2: I am grateful for the opportunity to review this paper. It is a very important and timely manuscript and I have no hesitation in saying it is exactly the kind/calibre of study I would expect to see in a journal such as PLoS Medicine.

The authors systematically examined parental smoking, alcohol, and caffeine use, alongside socioeconomic position, across four large cohorts and found that associations with child health were generally small, but more importantly found stronger evidence for socioeconomic influences than for parental behaviours. This is an ambitious and rigorous study, drawing on harmonised data and multiple causal inference approaches. The methods are presented with transparency and clear acknowledgement of limitations. The findings have important implications for both policy and future research, shifting focus beyond maternal pregnancy effects to partners and wider social determinants.

I also want to highlight the freely accessible EPoCH Explorer web app: I explored it myself and found it to be a genuinely useful (and quite fun!) tool for researchers, and I sincerely thank the authors for making it openly available.

Recommendations/points for consideration

1. Although it has long been suspected that socioeconomic conditions matter at least as much as parental behaviours in shaping child health, I am not aware of previous studies that have systematically tested this within a DOHaD framework; the authors should make clearer in the Introduction and Discussion that this is, to my knowledge, the first large-scale comparison of its kind (if it is not the first study pitting personal behaviours vs SES on this eoutcomes, it would be useful to know what other studies found)

2. The heavy reliance on complete-case analysis raises the possibility of selection bias if the missingness of data was not random - I think it would be important to acknowledge this explicitly as a limitation.

3 It would be useful to know the potential scale and impact of including non-biological partners in the analyses, particularly for the Mendelian randomisation work where biological paternity is central to the assumptions.

4. Genetic risk scores and instrument strength are far from my area of expertise, but nor will they be clear to most readers; a brief plain-language explanation of what a "weak instrument" means in practice, and why it matters for interpretation, would improve accessibility of the methods and results.

5. Not all analyses were multi-cohort (I think), and I found it difficult to determine which findings were based on pooled evidence and which on a single study; while this may not be central to the overall message, it is probably worth a sentence in the limitations section so readers are aware in case they miss this.

6. Certain important modifiers and mediators, such as breastfeeding, housing conditions, and vaccine uptake, are not discussed but may be relevant to interpreting both exposures and outcomes in this field (they are certainly hot topics) ; even if not available in the cohorts, a short note in the Limitations section would help readers place the findings in the wider context.

Overall, this is a substantial and well-conducted piece of work with high relevance for both research and policy. The findings may indeed be regarded as hugely influential because the headline (that socioeconomic conditions show stronger associations with child health than specific parental health behaviours) is important to many stakeholders and policymakers. Also the open resource provided will be of real value to the researchers in the field.

Ian Sinha, Alder Hey Children's Hospital, Liverpool, UK

Reviewer #3: Thank you for the opportunity to review this article. The EPOCH platform seems to provide a unique and ambitious tool that could expand the exploration of associations related to factors influencing the early stages of life course health.

The open access design offers transparency and the opportunity for the addition of further evidence from cohorts in contexts that vary from the four provided to date.

The explanation of the need for the tool and its strengths regarding additional analyses are well described.

The concept that SES is a complex construct correlated with health outcomes is not novel. DOHaD evidence explores the complexity of the interplay between genetic, environmental, socio-political, and lifestyle factors within and across generations.

Is the contested issue that the authors are pointing to the oversimplification of association evidence in a complex system? If so, maybe it would be sensible to make this clearer? Even smoking, which the EPoCH evidence clearly points to as causal, cannot be dissociated from SES.

There is no question that the tool has enormous potential and could be very useful in supporting deeper understanding of the complexity of factors associated with long-term health outcomes. The current study offers data up to age 8 - well before many of the later-life outcomes are emerging.

Some comments within the limitations on factors beyond the three important ones chosen could also be interacting within the system to influence outcomes. Maternal stress (which is of course associated with SEs) seems to be a clear omission - albeit challenging to measure.

It would also be helpful to see some comment on the limitations of the age to which the data reaches (age 8) re the associations of 'later life' health outcomes.

Reviewer #4: Many thanks for the opportunity to review this paper.

This is an ambitious, well-executed high-throughput DOHaD paper that will be very valuable to the field for two reasons: (i) it systematically benchmarks a large set of prenatal parental behaviours (maternal and partner) against multiple outcomes using harmonised multicohort analyses (with meta-analysis where possible), and (ii) it operationalises transparent evidence triangulation via an open, interactive web tool (EPoCH Explorer) with downloadable summary statistics and code.

The main scientific contribution is not a single novel causal claim, but a disciplined recalibration of expectations: most parental prenatal behaviour-child outcome associations are small and not robust after multiple testing, whereas socioeconomic position (SEP) shows consistently stronger associations across domains.

Minor points for consideration:

1.Clarify the inferential target (avoid "evidence of no effect" interpretation). The central message, which is limited widespread evidence for large prenatal behaviour effects, could be misread as disproving prenatal behavioural influences outcomes. Strengthen the framing in Abstract/Discussion to distinguish: (i) genuinely small effects after confounding control/multiple testing from (ii) null results driven by measurement error, limited partner data, or imprecision in specific exposure-outcome strata.

2. The authors note complete-case analysis and potential MNAR bias, but the scale of analyses amplifies concerns about selection (partner exposure availability, outcome follow-up). Could more detail or comment around missingness/attrition summary by cohort and domain be provided.

3. Partner exposure is a strong negative control for maternal intrauterine effects only when key assumptions hold. For outcomes where partner pathways are plausible (passive smoke exposure; shared household diet; parenting stress; postnatal continuation of behaviours), similarity does not necessarily imply "non-causal." Could he authors add an outcome typology and explicitly flag domains where partner negative controls are less clear?

4. FDR at 5% plus Cohen's D>0.2 is pragmatic, but the D threshold may be questioned across heterogeneous outcomes. Could the authors comment on sensitivity summaries using alternative thresholds (e.g., D>0.1) so aid justifying the approach?

5. The fixed-effects choice due to few cohorts is understandable, but readers may want clarity about heterogeneity (measurement, cohort composition). Could authors provide heterogeneity indicators and/or a small sensitivity comparison (fixed vs random) for key exposure-outcome pairs, or explicitly flag where heterogeneity is non-trivial and conclusions should be cautious.

6. The cohorts are predominantly European/Northern high-income settings; add a clearer statement on applicability to LMIC contexts and settings with different exposure distributions, SEP structures, and competing risks.

Any attachments provided with reviews can be seen via the following link: [LINK]

--------------------------------------------------------- ---

General editorial requests:

*Please upload any figures associated with your paper as individual TIF or EPS files with 300dpi resolution at resubmission; please read our figure guidelines for more information on our requirements: http://journals.plos.org/plosmedicine/s/figures. While revising your submission, we strongly recommend that you use PLOS's NAAS tool (https://ngplosjournals.pagemajik.ai/artanalysis) to test your figure files. NAAS can convert your figure files to the TIFF file type and meet basic requirements (such as print size, resolution), or provide you with a report on issues that do not meet our requirements and that NAAS cannot fix.

After uploading your figures to PLOS's NAAS tool - https://ngplosjournals.pagemajik.ai/artanalysis, NAAS will process the files provided and display the results in the "Uploaded Files" section of the page as the processing is complete.

If the uploaded figures meet our requirements (or NAAS is able to fix the files to meet our requirements), the figure will be marked as "fixed" above. If NAAS is unable to fix the files, a red "failed" label will appear above.

When NAAS has confirmed that the figure files meet our requirements, please download the file via the download option, and include these NAAS processed figure files when submitting your revised manuscript.

*We expect all researchers with submissions to PLOS in which author-generated code underpins the findings in the manuscript to make all author-generated code available without restrictions upon publication of the work. In cases where code is central to the manuscript, we may require the code to be made available as a condition of publication. Authors are responsible for ensuring that the code is reusable and well documented. Please make any custom code available, either as part of your data deposition or as a supplementary file. Please add a sentence to your data availability statement regarding any code used in the study, e.g. "The code used in the analysis is available from Github [URL] and archived in Zenodo [DOI link]" Please review our guidelines at https://journals.plos.org/plosmedicine/s/materials-software-and-code-sharing and ensure that your code is shared in a way that follows best practice and facilitates reproducibility and reuse. Because Github depositions can be readily changed or deleted, we encourage you to make a permanent DOI'd copy (e.g. in Zenodo) and provide the URL.

* Data availability statement: Please include a statement on the criteria that researchers need to fulfill in order to be granted access to the data.

* Ethics statement: I see that for each cohort there was consent from an ethics committee. However, please provide an ethics statement in the method on whether this particular study is approved by an ethics committee. You can include in this statement that the descriptions/ethics approval for each cohort can be found in the supplements. Please also ensure that for each cohort you describe whether participants provided oral or written consent.

FORMATTING - GENERAL

*Abstract: Please structure your abstract using the PLOS Medicine headings (Background, Methods and Findings, Conclusions). Please combine the Methods and Findings sections into one section.

*At this stage, we ask that you include a short, non-technical Author Summary of your research to make findings accessible to a wide audience that includes both scientists and non-scientists. The Author Summary should immediately follow the Abstract in your revised manuscript. This text is subject to editorial change and should be distinct from the scientific abstract. Ideally each sub-heading should contain 2-3 single sentence, concise bullet points containing the most salient points from your study. In the final bullet point of 'What Do These Findings Mean?', please include the main limitations of the study in non-technical language. Please see our author guidelines for more information: https://journals.plos.org/plosmedicine/s/revising-your-manuscript#loc-author-summary.

*Please express the main results with 95% CIs as well as p values. When reporting p values please report as p<0.001 and where higher as the exact p value p=0.002, for example. Throughout, suggest reporting statistical information as follows to improve clarity for the reader "22% (95% CI [13%,28%]; p</=)". Please be sure to define all numerical values at first use.

*Please include page numbers and line numbers in the manuscript file. Use continuous line numbers (do not restart the numbering on each page).

*Please cite the reference numbers in square brackets. Citations should precede punctuation.

FIGURES AND TABLES

*Please provide titles and legends for all figures and tables (including those in Supporting Information files).

*Please define all abbreviations used in each figure/table (including those in Supporting Information files).

SUPPLEMENTARY MATERIAL

*Please note that supplementary material will be posted as supplied by the authors. Therefore, please amend it according to the relevant comments outlined here.

*Please cite your Supporting Information as outlined here: https://journals.plos.org/plosmedicine/s/supporting-information

REFERENCES

*PLOS uses the numbered citation (citation-sequence) method and first six authors, et al.

*Please ensure that journal name abbreviations match those found in the National Center for Biotechnology Information (NCBI) databases (http://www.ncbi.nlm.nih.gov/nlmcatalog/journals), and are appropriately formatted and capitalised.

*Where website addresses are cited, please include the complete URL and specify the date of access (e.g. [accessed: 12/06/2023]).

*Please also see https://journals.plos.org/plosmedicine/s/submission-guidelines#loc-references for further details on reference formatting.

[STUDY TYPE-SPECIFIC REQUESTS - DELETE SECTIONS AS NECESSARY]

OBSERVATIONAL STUDIES

*Abstract: Please include the study design, population and setting, number of participants, years during which the study took place (enrollment and follow up), length of follow up, and main outcome measures.

*Please state in the Methods section whether the study had a prospective protocol or analysis plan. If a prospective analysis plan (from your funding proposal, IRB or other ethics committee submission, study protocol, or other planning document written before analyzing the data) was used in designing the study, please include the relevant document(s) with your revised manuscript as a Supporting Information file to be published alongside your study and cite it in the Methods section. A legend for this file should be included at the end of your manuscript. If no such document exists, please make sure that the Methods section transparently describes when analyses were planned, and when/why any data-driven changes to analyses took place. Changes in the analysis, including those made in response to peer review comments, should be identified as such in the Methods section of the paper, with rationale.

MENDELIAN RANDOMIZATION STUDIES

*Please ensure that the study is reported according to the STROBE-MR guideline (https://www.equator-network.org/reporting-guidelines/strobe/) and include the completed STROBE-MR checklist as Supporting Information. Please add the following statement, or similar, to the Methods: "This study is reported as per the Strengthening the Reporting of Observational Studies in Epidemiology (STROBE) guideline, specific for mendelian randomization (S1 Checklist)." When completing the checklist, please use section and paragraph numbers, rather than page numbers.

*In the Introduction, please describe the exposure and the evidence for a potential causal relationship between exposure and outcome.

*In the Methods, please explicitly state the 3 core instrumental variable assumptions for the main analysis (relevance, independence, and exclusion restriction), as well assumptions for any additional or sensitivity analysis.

*In the Methods, please describe the MR estimator (e.g., 2-stage least squares, Wald ratio) and related statistics. Detail the included covariates and, in case of 2-sample MR, whether the same covariate set was used for adjustment in the 2 samples.

*If you are presenting an instrumental variable estimate, please compare this to the conventional observational estimate.

*Report the associations between genetic variant and exposure and between genetic variant and outcome, preferably on an interpretable scale.

*Report MR estimates of the relationship between exposure and outcome and the measures of uncertainty from the MR analysis, on an interpretable scale, such as odds ratio or relative risk per SD difference.

*If relevant, please consider translating estimates of relative risk into absolute risk for a meaningful time period.

*Please consider including plots to visualize results (e.g., forest plot, scatterplot of associations between genetic variants and outcome vs between genetic variants and exposure).

# Author comment: Author response to Decision Letter 2

**DOI:** 10.1371/journal.pmed.1005153.r003

17 Apr 2026

Attachment Submitted filename: PlosMedicine_ResponseToReviewers.docx

# Peer review: Decision Letter 2

**DOI:** 10.1371/journal.pmed.1005153.r004

**Authors:** Richard Turner, Suzanne De Bruijn, Suzanne De Bruijn

26 May 2026

Dear Dr. Sharp,

Thank you very much for re-submitting your manuscript "Exploring parental prenatal influences on child health: a multicohort study and data visualisation tool" (PMEDICINE-D-25-02845R2) for review by PLOS Medicine.

I have discussed the paper with my colleagues and the academic editor and it was also seen again by 3 reviewers. I am pleased to say that provided the remaining editorial and production issues are dealt with we are planning to accept the paper for publication in the journal.

The remaining issues that need to be addressed are listed at the end of this email. Any accompanying reviewer attachments can be seen via the link below. Please take these into account before resubmitting your manuscript:

[LINK]

***Please note while forming your response, if your article is accepted, you may have the opportunity to make the peer review history publicly available. The record will include editor decision letters (with reviews) and your responses to reviewer comments. If eligible, we will contact you to opt in or out.***

In revising the manuscript for further consideration here, please ensure you address the specific points made by each reviewer and the editors. In your rebuttal letter you should indicate your response to the reviewers' and editors' comments and the changes you have made in the manuscript. Please submit a clean version of the paper as the main article file. A version with changes marked must also be uploaded as a marked up manuscript file.

Please also check the guidelines for revised papers at http://journals.plos.org/plosmedicine/s/revising-your-manuscript for any that apply to your paper. If you haven't already, we ask that you provide a short, non-technical Author Summary of your research to make findings accessible to a wide audience that includes both scientists and non-scientists. The Author Summary should immediately follow the Abstract in your revised manuscript. This text is subject to editorial change and should be distinct from the scientific abstract.

In addition to these revisions, you may need to complete some formatting changes, which you will receive in a follow up email. A member of our team will be in touch with a set of requests shortly. If you do not receive a separate email within a few days, please assume that checks have been completed, and no additional changes are required.

We expect to receive your revised manuscript within 1 week. Please email us (plosmedicine@plos.org) if you have any questions or concerns.

Please review your reference list to ensure that it is complete and correct. If you have cited papers that have been retracted, please include the rationale for doing so in the manuscript text, or remove these references and replace them with relevant current references. Any changes to the reference list should be mentioned in the rebuttal letter that accompanies your revised manuscript.

Please note, when your manuscript is accepted, an uncorrected proof of your manuscript will be published online ahead of the final version, unless you've already opted out via the online submission form. If, for any reason, you do not want an earlier version of your manuscript published online or are unsure if you have already indicated as such, please let the journal staff know immediately at plosmedicine@plos.org.

If you have any questions in the meantime, please contact me or the journal staff on plosmedicine@plos.org.

We look forward to receiving the revised manuscript by Jun 02 2026 11:59PM.

Sincerely,

Suzanne De Bruijn, PhD

Associate Editor

PLOS Medicine

plosmedicine.org

------------------------------------------------------------

Requests from Editors:

GENERAL REQUESTS:

* Please confirm that your title complies with PLOS Medicine's style. Your title must be nondeclarative and not a question. It should begin with main concept if possible. "Effect of" should be used only if causality can be inferred, i.e., for an RCT. Please place the study design ("A randomized controlled trial," "A retrospective study," "A modelling study," etc.) in the subtitle (ie, after a colon).

* Please confirm that your abstract complies with our requirements, including format (three sections: Background, Methods and Findings, and Conclusions) and providing all the information relevant to this study type https://journals.plos.org/plosmedicine/s/submission-guidelines#loc-abstract

* Please ensure that all abbreviations are defined at first use throughout the text.

* Please confirm that all numbers presented in the abstract are present and identical to numbers presented in the main manuscript text.

* Please disclose any use of AI tools and technologies to the study or article content in the Methods, including the name(s) of any tool(s) used and a description of their use. Please see our guidelines for more information: https://journals.plos.org/plosmedicine/s/ethical-publishing-practice#loc-artificial-intelligence-tools-and-technologies.

* Please review your text for claims of novelty or primacy (e.g. 'for the first time') and remove this language. In addition, please check that any use of statistical terms (such as trend or significant) are supported by the data, and if not please remove them.

* Please remove the 'conclusions' subheading from the discussion. Please also remove any other subheadings from the discussion.

* Statistical reporting: Please revise throughout the manuscript, including tables and figures.

- Please report statistical information as follows to improve clarity for the reader ""22% (95% CI [13,28]; p</=)"".

- Please separate upper and lower bounds with commas instead of hyphens as the latter can be confused with reporting of negative values.

- Please repeat statistical definitions (HR, CI etc.) for each set of parentheses.

* Line 184-185: please check if this sentence is correct, and modify if necessary: “caffeine from tea, coffee, cola, and any of those sources” .

ABSTRACT:

* In the last sentence of the Abstract Methods and Findings section, please describe the main limitation(s) of the study's methodology.

DATA AVAILABILITY:

*Thank you for providing the used code, as well as a Zenodo link. We need a permanent record of all the data. As the website could at some point be taken down for any reason, can you:

1) Confirm that all results can be generated with the provided code?

2) Can you provide the summary statistics in a different way, instead of only on the EPoCH Explorer? This could for instance be as a supplemental file, or be included in the code/Zenodo links?

*Could you include your statement on Code availability in your Data availability statement (in the MS and the metadata)?

ETHICS STATEMENT:

*Thank you for including your ethics statement. do you have approval numbers from the ALSPAC, BIB and MoBa executive board? If yes, please include these in your ethics statement.

FIGURES

* When a p value is given, please specify the statistical test used to determine it in the legend.

OBSERVATIONAL STUDIES

* Thank you for including your analysis plan as a supplement. Am I correct that this was a prospective plan? Please state this (either way) early in the Methods section.

a) If a prospective analysis plan (from your funding proposal, IRB or other ethics committee submission, study protocol, or other planning document written before analyzing the data) was used in designing the study, please include the relevant prospectively written document with your revised manuscript as a Supporting Information file to be published alongside your study, and cite it in the Methods section. A legend for this file should be included at the end of your manuscript.

b) If no such document exists, please make sure that the Methods section transparently describes when analyses were planned, and when/why any data-driven changes to analyses took place.

c) In either case, changes in the analysis-- including those made in response to peer review comments-- should be identified as such in the Methods section of the paper, with rationale.

* Thank you for including the STROBE checklist. Could you provide this as a separate file, rather than as part of the supplementary material?

Comments from Reviewers:

Reviewer #1: I thank the authors for their responses to my comments. These have been addressed satisfactorily, and I have no further comments.

Reviewer #2: Thank you for making these changes, which I think improve the paper - I am happy to recommend to the Editorial Team that the paper is ready to accept in my opinion.

Reviewer #4: The authors have very carefully and thoughtfully addressed both major and minor comments raised. The paper is certainly improved and is more precise. No further comments from me.

Any attachments provided with reviews can be seen via the following link:

[LINK]

# Author comment: Author response to Decision Letter 3

**DOI:** 10.1371/journal.pmed.1005153.r005

1 Jun 2026

Attachment Submitted filename: R2 Response.docx

# Editor report: Decision Letter 3

**DOI:** 10.1371/journal.pmed.1005153.r006

**Authors:** Richard Turner, Suzanne De Bruijn, Suzanne De Bruijn, Suzanne De Bruijn

5 Jun 2026

Dear Dr Sharp,

On behalf of my colleagues and the Academic Editor, Mark Tomlinson, I am pleased to inform you that we have agreed to publish your manuscript "Exploring parental prenatal influences on child health: a multicohort study and data visualisation tool" (PMEDICINE-D-25-02845R3) in PLOS Medicine.

Before your manuscript can be formally accepted we have 2 remaining requests:

*Thank you for providing the STROBE checklist as separate files; Please provide legends for each of these files, and update their citation in the methods section.

* Data availability statement: Please replace the phrase ‘bona fide researchers’. We suggest: “Access to individual level data is available, subject to review and approval."

Furthermore, you will need to complete some formatting changes, which you will receive in a follow up email. Please be aware that it may take several days for you to receive this email; during this time no action is required by you. Once you have received these formatting requests, please note that your manuscript will not be scheduled for publication until you have made the required changes.

In the meantime, please log into Editorial Manager at http://www.editorialmanager.com/pmedicine/, click the "Update My Information" link at the top of the page, and update your user information to ensure an efficient production process.

PRESS

We frequently collaborate with press offices. If your institution or institutions have a press office, please notify them about your upcoming paper at this point, to enable them to help maximise its impact. If the press office is planning to promote your findings, we would be grateful if they could coordinate with medicinepress@plos.org. If you have not yet opted out of the early version process, we ask that you notify us immediately of any press plans so that we may do so on your behalf.

We also ask that you take this opportunity to read our Embargo Policy regarding the discussion, promotion and media coverage of work that is yet to be published by PLOS. As your manuscript is not yet published, it is bound by the conditions of our Embargo Policy. Please be aware that this policy is in place both to ensure that any press coverage of your article is fully substantiated and to provide a direct link between such coverage and the published work. For full details of our Embargo Policy, please visit http://www.plos.org/about/media-inquiries/embargo-policy/.

Thank you again for submitting to PLOS Medicine. We look forward to publishing your paper.

Sincerely,

Suzanne De Bruijn, PhD

Associate Editor

PLOS Medicine
